# MALDI-TOF MS-Based Lipidomic Profile of Honey and Bee Pollen

**DOI:** 10.1021/acsagscitech.5c00883

**Published:** 2025-11-06

**Authors:** Ana Jano, Adrián Fuente-Ballesteros, Jesús A. Tapia, Silvia Valverde, Ana M. Ares, José Bernal

**Affiliations:** † Analytical Chemistry Group (TESEA), I. U. CINQUIMA, Faculty of Sciences, 16782 University of Valladolid, Valladolid 47011, Spain; ‡ Department of Statistics and Operations Research, Faculty of Sciences, University of Valladolid, Valladolid 47011, Spain

**Keywords:** MALDI-TOF MS, lipid profiling, lipidomics, food chemistry, bee product, food authentication, metrics, green analytical chemistry, biomarker, principal component analysis, PCA

## Abstract

The increasing demand for bee-derived products such as honey and bee pollen has led to a rise in adulteration and mislabeling, making it essential to develop reliable tools for authentication. Lipids, which are found in both matrices, are potential biomarkers for tracing their origin and may be used for detecting fraud. In this work, a solid–liquid extraction using hexane:isopropanol (10:1, v/v) followed by matrix-assisted laser desorption/ionization time-of-flight mass spectrometry (MALDI-TOF MS) was optimized. The method was applied for tentative lipid screening of 15 honeys and 13 bee pollens showing a total number of lipids above 700, including fatty acyls, glycerolipids, glycerophospholipids, sphingolipids, and sterol lipids. For the first time, a principal component analysis was carried out for botanical and geographical origin, classifying most of the samples correctly. Additionally, the method was categorized as green (environmentally friendly) and blue (practical).

## Graphical abstract

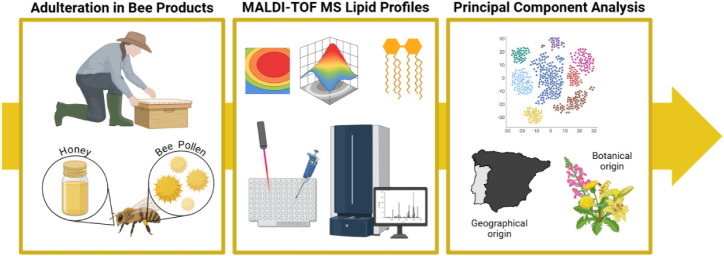

## 1 Introduction

In recent years, bee-derived products, such as honey and bee pollen, have attracted increasing attention due to their rich composition in bioactive compounds with recognized health benefits.^1,2^ Their chemical composition is shaped by multiple factors, with botanical and geographical origins being the most influential.^3–5^ Nonetheless, environmental conditions and beekeeping practices also add to the variability observed in these products. Because of this strong correlation between composition and origin, chemical profiling of these products has long served as a reliable means to assess authenticity, preventing false origin labeling, that is, fraud.^6^ Recently, a new regulation related to these products has been published: it being the Royal Decree 68/2025, dated February 4, which amends Royal Decree 1049/2003 of August first, approving the quality standard for honey. Determining the floral source of both bee pollen and honey necessitates microscopic examination of pollen grains, a technique known as melissopalynology,^7^ a method which demands highly trained specialists. It can also be complemented by both sensory evaluation and physicochemical characterization, but this often lacks specificity, and cannot be reliably generalized across all bee pollen and honey types.^8^ As a result, there has been growing interest in developing faster yet dependable strategies for distinguishing honeys according to their floral origin. These alternative methodologies typically involve the assessment of multiple chemical parameters combined with multivariate statistical tools, such as elemental analyzer/liquid chromatography-isotope ratio mass spectrometry, high-performance anion exchange chromatography with pulsed amperometric detection, or proton nuclear magnetic resonance spectroscopy.^9^ These can be applied in order to detect biomarkers. A biomarker is a molecule whose presence, composition, or abundance reflects authenticity-related attributes of a biological system such as species, geographical origin, processing history, or freshness, since these characteristics are genetically determined and specifically expressed through the organism’s metabolism.^10^ So, fully determining the composition is the key to detecting food fraud. In this context, bee pollen is a complex matrix containing essential amino acids, antioxidants, vitamins, and lipids.^1^ The latter, in particular, are crucial as energy reserves and structural components, needed for pollen grain viability and germination. Depending on its botanical source, lipid content in bee pollen can vary significantly, ranging from 1% to 20% of its dry weight.^11^ Honey, while mainly composed of carbohydrates (typically between 60% and 85%) and water (12–23%), also contains minor compounds such as organic acids, minerals, vitamins, enzymes, proteins, amino acids, and lipids.^12^ This last fraction has been found only in trace amounts—around 0.04%—and includes glycerides, sterols, phospholipids, and fatty acids such as palmitic, oleic, lauric, myristic, stearic, and linoleic acids.^11^ Notably, the natural presence of bee pollen grains in honey may lead to the incidental incorporation of additional compounds typical of the bee pollen matrix. Other constituents of honey and bee pollen, such as amino acids^13^ and glucosinolates,^14^ have already been proposed as effective biomarkers for determining botanical and/or geographical origin, highlighting the value of these matrices for product authentication. In this context, lipids, which are also present in both of these bee matrices, emerge as promising candidates for biomarkers due to their structural diversity and sensitivity to environmental and botanical factors.

Lipidomics are gaining prominence in the areas of food authentication and the detection of adulteration.^15^ Various analytical techniques have been employed to profile lipid compositions in different food matrices. For instance, ultrahigh-performance liquid chromatography (UHPLC) coupled with different mass spectrometry detectors, has been applied to uncover fraudulent practices in green tea,^15^ or to differentiate toxic from nontoxic mushroom species through the identification of lipid markers.^16^ Other matrices analyzed with this technique are fish fillets^17^ or coffee beans.^18^ However, limited information about lipids in bee products is available. Gas chromatography coupled with flame ionization detection (GC-FID) has been used to provide a qualitative analysis of fatty acids in honey, bee pollen, bee bread, and propolis.^19^ Matrix-assisted laser desorption/ionization time-of-flight mass spectrometry (MALDI-TOF MS) has also been used for lipid fingerprinting and microbiome characterization.^20^ It exhibits good characteristics for a fast and sensitive generation of tentative molecular profiles with minimal sample prep optimization, which is aligned with green analytical chemistry (GAC) principles.^21^ This feature is particularly advantageous for preliminary studies, as it provides a broad overview of the sample composition and facilitates the identification of a larger number of potential biomarkers. Once the initial pool of compounds is narrowed down, targeted and quantitative techniques could be applied for more focused validation. This technique has been already applied to detect lipids in a variety of food matrices such as milk,^22^ oil,^23^ rice,^24^ and lentils,^25^ being 2,5-dihydroxybenzoic acid (DHB) and chloroform the MALDI matrix and extraction solvent most frequently reported, respectively (see Supporting Information Table 1S). In the aforementioned studies, the analytes belonged to five major lipid families included in the Lipid Metabolites and Pathways Strategy database (LIPID MAPS):^26^ fatty acyls, glycerolipids, glycerophospholipids, sphingolipids, and sterol lipids.

In this work, we propose an optimized sample preparation protocol followed by MALDI-TOF MS analysis for the tentative profiling of lipids in honey and bee pollen. The advantages of using lipids as biomarkers stem from the simplicity and efficiency of the workflow. Sample preparation is minimal, MALDI-TOF MS supports rapid, high-throughput analysis, and the method can deliver an overview of the sample’s composition. This approach enables both the generation of a global fingerprint and, when necessary, the targeted monitoring of specific lipids of interest. This approach aims to support the discrimination of both botanical and geographical origins based on the full lipid profile in the sample. To date, only one study^27^ has reported lipid profiling in bee pollen and none in honey, underscoring the novelty and relevance of the present research. Understanding the lipid profile of bee-derived products not only contributes to their authentication but also provides insights into their nutritional quality and potential health benefits.

## 2 Materials and Methods

### 2.1 Reagents and Instrumentation

All solvents (tetrahydrofuran, hexane, isopropanol, methanol, chloroform, heptane, cyclohexane, and dichloromethane) were of chromatographic grade and were obtained from Sigma-Aldrich (Madrid, Spain), Carlo Erba Reagents, and Panreac Química (Barcelona, Spain). The calibrant for MALDI-TOF, polyethylene glycol (PEG 1000, 10 mg/mL), was purchased from Sigma-Aldrich (Steinheim, Germany). MALDI matrices such as 3′,5′-dimethoxy-4-hydroxyacetophenone (DHAP), dithranol (DIT), trans-2-[3-(4-*tert*-butylphenyl)-2-methyl-2-propenylidene]­malononitrile (DCTB), 2,5-dihydroxybenzoic acid (DHB), cinnamic chloride (CC), 4-hydroxy-3-methoxycinnamaldehyde (HMCA) and 1-amino-2-naphthol-4-sulfonic acid (AANS), and ionizing agents (silver and sodium trifluoroacetate, AgTFA and NaTFA) were obtained from Sigma-Aldrich (Steinheim, Germany).

Laboratory equipment included an EA-240 analytical balance (Mettler Toledo, Darmstadt, Germany), ThermoFisher micropipettes (Waltham, MA, USA), and Eppendorf tubes (Labbox SL, Barcelona, Spain). Sample mixing and processing were performed using a Heidolph vortex mechanical mixer (Schwabach, Germany), a Vibromatic mechanical shaker and an ultrasound bath both supplied by J.P. Selecta S.A. (Barcelona, Spain). Centrifugation was conducted using an Eppendorf refrigerated benchtop centrifuge (Hamburg, Germany).

### 2.2 Samples

A total of 28 samples were analyzed (see Table 1), including 15 honey and 13 bee pollen samples. The honey samples were from different botanical origins determined through melissopalynological analysis such as multifloral, chestnut (*Castanea sativa*), sainfoin (*Onobrychis viciifolia*), rapeseed (*Brassica napus*), lavender (*Lavandula* *spp*.), clover (*Trifolium* *spp*.), and heather (*Erica* spp.) honeys. Similarly, the bee pollen samples consisted of 10 multifloral and 3 chestnut bee pollen specimens. All samples were collected from different geographic regions across Spain. Honey samples were stored at −20 °C until analysis to preserve their lipid composition. Bee pollen samples were dried in an oven at 45 °C for 24 h, ground individually using a mill, pooled to ensure optimal sample homogeneity and subsequently stored in a dry-seal vacuum desiccator at room temperature until analysis.^28^ Three replicates (subsamples) of each sample, which were analyzed in triplicate, were examined to determine the lipid content.

**Table 1:** Botanical and Geographical Origins of the Analyzed Honey and Bee Pollen Samples^a^,^b^

Honey Samples			Bee Pollen Samples		
Code	Botanical Origin	Geographical Origin	Code	Botanical Origin	Geographical Origin
H1	Multifloral	Salamanca	P1	Multifloral	Spain
H2	Multifloral	Spain	P2	Multifloral	Spain
H3	Chestnut	Galicia	P3	Multifloral	Granada
H4	Chestnut	Ávila	P4	Multifloral	Spain
H5	Multifloral	Ávila	P5	Chestnut	Spain
H6	Multifloral	Cuenca	P6	Multifloral	Galicia
H7	Multifloral	Palencia	P7	Multifloral	Galicia
H8	Sainfoin	Palencia	P8	Multifloral	Spain
H9	Rapeseed	Palencia	P9	Multifloral	Spain
H10	Lavender	Cuenca	P10	Multifloral	Spain
H11	Trifolium	Palencia	P11	Chestnut	Spain
H12	Lavender	Cuenca	P12	Multifloral	Spain
H13	Heather	Palencia	P13	Chestnut	Galicia
H14	Heather	Palencia			
H15	Multifloral	Palencia			

a H, honey.

b P, bee pollen.

### 2.3 Sample Treatment

The sample preparation protocols to extract lipids from honey and bee pollen were designed to be simple, reproducible, and fast, and are largely comparable. Both methods are illustrated in Figure 1.

**Figure 1:**
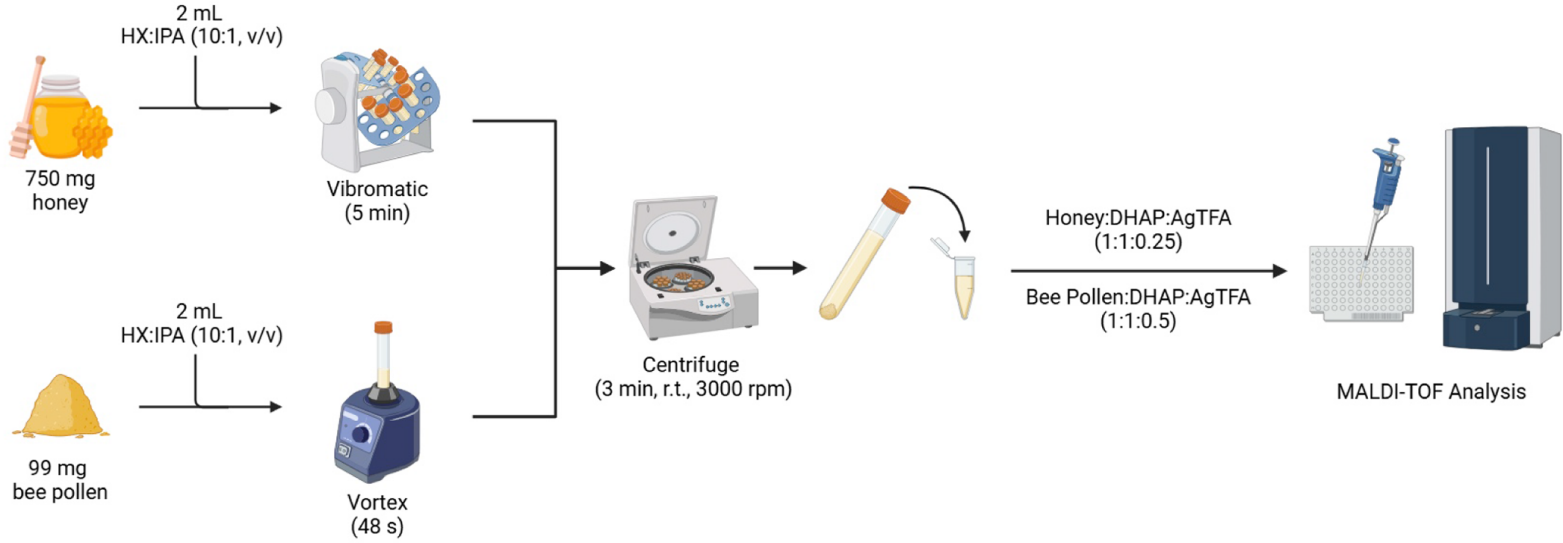
Sample preparation workflows for honey and bee pollen.

#### 2.3.1 Honey Samples

Briefly, 750 mg of homogenized honey were weighed into a 10 mL centrifuge tube. A volume of 2 mL of a mixture of hexane:isopropanol (10:1 v/v) was then added. The mixture was shaken using a Vibromatic shaker for 5 min and the samples were centrifuged at 10 000 rpm for 3 min at room temperature, allowing the solid phase to settle at the bottom of the tube. The resulting supernatant was decanted and collected for subsequent MALDI-TOF MS analysis.

#### 2.3.2 Bee Pollen Samples

Briefly, 99 mg of homogenized bee pollen were weighed in a 10 mL centrifuge tube, followed by addition of 2 mL of a hexane:isopropanol (10:1 v/v). The mixture was agitated for 48 s in a vortex device and subsequently centrifuged at 10 000 rpm for 3 min at room temperature. The supernatant was then decanted and used for MALDI-TOF MS lipid profiling.

### 2.4 MALDI-TOF Conditions

Lipid profiling was performed using MALDI-TOF MS in positive reflector mode. The instrument (Autoflex maX, Bruker Daltonics, Bremen, Germany) was equipped with a laser operating at a wavelength of 335 nm, a frequency of 1000 Hz and using a 5 GS/s digitizer. The dynamic range was set from *m*/*z* 200 to 2200, and a suppression gate up to *m*/*z* 350 was applied to prevent detector saturation by low-mass ions, especially from the MALDI matrix. The laser power was set to 50% for both honey and bee pollen samples. PEG 1000 in tetrahydrofuran (10 mg mL^–1^) was used as a calibrant with DCTB as the MALDI matrix (PEG 1000:DCTB, 4:10, v/v), and the calibration error was of 0.3 Da. The MALDI matrix used for the analyses was DHAP in dichloromethane (15 mg mL^–1^), and the ionizing agent was an AgTFA solution in tetrahydrofuran (10 mg mL^–1^). For honey samples, the sample-to-matrix-to-ionizing agent ratio was 1:1:0.25 (v/v/v), whereas for bee pollen the ratio was 1:1:0.5 (v/v/v). To improve reproducibility and ensure more homogeneous crystallization, the sample, MALDI matrix, and ionizing agent were mixed in an Eppendorf tube immediately prior to deposition of 0.5 μL of the mixture onto the MALDI plate. Spectra acquisition and processing were performed under standardized conditions. Lipids were tentatively identified by comparing experimental *m*/*z* values to entries in the LIPID MAPS database^29^ with a mass accuracy threshold of less than ± 0.05 Da. Lipid signals were assigned based on the database entry with the lowest mass error relative to the experimental *m*/*z* value. To improve identification reliability and minimize false positives arising from MALDI matrix-related signals, spectra were also acquired from the MALDI matrix, prepared with the same matrix-to-ionizing agent ratio used in the sample mixtures. This allowed for the subtraction of MALDI matrix-derived peaks from the lipid profiles of each sample. MALDI matrix-only controls were recorded freshly on each analytical day to account for possible day-to-day variability.

### 2.5 Statistical Analysis

Design of experiments (DoE) was conducted using MODDE 13.1 (Sartorius Stedim Data Analytics AB). Multivariate statistical analyses were performed to evaluate the discriminative power of the lipid profiles obtained from honey and bee pollen samples. Principal component analysis (PCA) was carried out using the PROC CANDISC procedure in SAS software, version 9.4 (SAS Institute Inc., Cary, NC, USA), to assess the separation between groups based on botanical and geographical origin.

## 3 Results and Discussion

### 3.1 Preliminary Tests

To establish optimal extraction conditions for lipid profiling of honey and bee pollen, preliminary experiments were conducted comparing several solvents and solvent mixtures in different proportions. These are very different matrices both in terms of structure and composition, so the sample treatment might differ and they cannot be considered as equivalent, although that would be optimal. Plus, both are very complex and can lead to interferences, which would be specially inconvenient for untargeted analyses, and so, an initial extraction of the compounds of interest is crucial. Both solid–liquid and liquid–liquid extraction approaches were evaluated to identify conditions yielding the highest number of mass spectral signals attributable to lipids, as confirmed by database matching in LIPID MAPS.^29^ Solvents most used in the literature included chloroform,^30^ methanol^31^ or mixtures of the both of them,^32^ as well as some other organic solvents such as hexane^23^ or dichloromethane.^33^ These and some others were tested as can be seen in Table 2. Initial tests according to available sample amount used 20 min ultrasonic extraction with a solvent volume of 2 mL and sample amounts of 500 mg for honey and 10 mg for bee pollen. It must be mentioned that in every case, bee pollen had to be grinded in order to be able to access the lipids in the inside of the grains, as bee pollen composition varies from the outer layers to the inner ones.^34^ Furthermore, there can be slight differences between different bee pollen grains in the same lot, so this can provide more representative results overall. After the extraction, centrifugation was needed in order to separate the solid residue of the samples and facilitate decantation. This was set for 3 min at 10 000 rpm as it provided adequate separation. Liquid–liquid extractions were performed only for honey and consisted in dissolving the sample in 2 mL of water before adding the organic solvent. The results indicated that these extractions were less efficient than solid–liquid ones. Detection in these experiments was performed in positive mode as this allowed for the detection of a broader range of lipid families and therefore has been more usually employed in similar studies.^23,35,36^ No ionizing agent was used, and DCTB was selected as the MALDI matrix, in a 1:1 ratio with the sample. Among the tested solvents, the mixture hexane:isopropanol (10:1, v/v) provided the most numerous lipid-related signals with minimal interference from MALDI matrix peaks. It must also be mentioned that higher proportions of isopropanol, as well as other alcohols, led to spreading of the extractant across the MALDI target wells, resulting in cross-contamination between samples. It is also worth noting that the mass range used in the literature typically extends from *m*/*z* 200 to 2000,^22,37^ with only a few studies extending the upper limit to *m*/*z* 4000 or 5000.^38,39^ In this work, a range from *m*/*z* 350 to 2200 was selected to maximize lipid detection while remaining within a range comparable to most published articles.

**Table 2:** Number of Lipids Detected for Each Extraction Technique and Solvent Evaluated for Honey and Bee Pollen^a^,^b^,^c^,^d^,^e^,^f^,^g^

Extraction technique	Solvent(s)	Ratio (v/v)	No. of Lipids (Honey)	No. of Lipids (Bee Pollen)
S-L	MeOH		23	28
S-L	CHCl_3_		32	24
L-L	CHCl_3_		28	
S-L	IPA		19	17
S-L	HX		39	33
L-L	HX		31	
S-L	CHCl_3_:MeOH	1:1	18	21
S-L	CHCl_3_:MeOH	2:1	27	17
S-L	CHCl_3_:MeOH	5:1	29	14
S-L	CHCl_3_:MeOH	10:1	23	14
S-L	HX:IPA	1:1	23	25
S-L	HX:IPA	2:1	31	26
S-L	HX:IPA	5:1	35	30
S-L	HX:IPA	10:1	43	34
S-L	Heptane		19	19
L-L	Heptane		13	
S-L	CX		29	23
L-L	CX		27	
S-L	DCM		27	21
L-L	DCM		24	

a S-L, solid–liquid extraction.

b MeOH, methanol.

c L-L, liquid–liquid extraction.

d IPA, isopropanol.

e HX, hexane.

f CX, cyclohexane.

g DCM, dichloromethane.

Moreover, several MALDI matrices, namely DIT, DCTB, DHB, CC, DHAP, and AANS, were evaluated to identify the optimal MALDI matrix for selective detection of lipid signals, as they are representative compounds belonging to the families most used for this purpose (benzoic acid derivatives, cinnamic acid derivatives, heterocycles and others).^40^ Some of them were discarded in terms of their performance. First, AANS was excluded due to spontaneous and highly irregular precipitation, in some stances even before deposition, varying concentration of the MALDI matrix solution. Other options such as DIT and CC, produced few background signals, which is desirable; however, they yielded negligible signals from the sample, often limited to MALDI matrix-derived peaks or background noise. Adversely, DCTB generated a high number of signals in the *m*/*z* 400–1000 range, interfering with sample signal detection and interpretation. DHB which has been more widely reported (see Supporting Information Table 1S), gave good results in positive mode, but both this MALDI matrix and HMCA showed fewer lipid signals in the samples compared to DHAP (see Supporting Information Figure 1S). Therefore, the selected MALDI matrix was the latter, as it provided the best results, crystallizing homogeneously and producing fewer signals at higher *m*/*z* values. Most lipid-related signals with DHAP were observed below *m*/*z* 400, facilitating clear lipid profiling with minimal MALDI matrix overlapping over this value. The signals below were therefore not considered in the analysis.

In the reviewed literature, ionizing agents are not usually applied, but some works opted for introducing sodium (NaTFA or NaCl)^41–43^ cations for a better performance, and silver is a well-known cation in this matter as well.^44^ For its evaluation, two ionizing agents (AgTFA and NaTFA) were tested, each dissolved in methanol and tetrahydrofuran. NaTFA produced very few sample-related signals in both solvents, suggesting poor interaction or ionization efficiency under the tested conditions. In contrast, AgTFA performed well in terms of improving the intensity of the signals, and therefore, the number of detected peaks. AgTFA in tetrahydrofuran was selected as it facilitated more uniform spot deposition on the MALDI target plate, given that it has higher surface tension and extends less around the plate.

Furthermore, in order to improve reproducibility, the sample, the MALDI matrix, and the ionizing agent were mixed thoroughly in a 0.5 mL Eppendorf tube prior to deposition of 0.5 μL on the target plate, rather than sequentially depositing each component directly into the well. Mixing was performed using the micropipette tip inside the tube, ensuring homogeneous suspension and preventing variability caused by uneven crystallization and poor spreading, especially of the ionizing agent. This modification significantly enhanced spectral reproducibility across replicate analyses.

### 3.2 Optimization of the Detection

Negative ion mode was initially tested as part of the analytical procedure; however, no signals were detected and as a result, no further optimization was pursued in this mode, and the corresponding data are not shown in this work. Following this, once the MALDI matrix (DHAP), ionizing agent (AgTFA) and extractant solvent (hexane: isopropanol, 10:1, v/v) were set, the detection parameters including laser power and sample:matrix:ionizing agent ratios were optimized (see Table 3). The evaluation was based on the total number of signals assigned to lipid species after subtracting those detected in the MALDI matrix-only controls, and reproducibility was assessed by consecutive measurements.

**Table 3:** Number of Lipids Detected for Each Condition for MALDI-TOF MS Analysis in Honey and Bee Pollen Samples, Using DHAP in DCM (15 mg mL^–1^) as the MALDI Matrix and AgTFA in THF (10 mg mL^–1^) as the Ionizing Agent

Laser Intensity (%)	Sample:Matrix:Agent (v/v/v)	No. of Lipids (Honey)	No. of Lipids (Bee Pollen)
30	1:1:0.25	13	8
30	1:1:0.5	11	9
30	1:1:1	12	28
50	1:1:0.25	45	36
50	1:1:0.5	40	41
50	1:1:1	42	33
60	1:1:0.25	38	36
60	1:1:0.5	40	32
60	1:1:1	37	29

In terms of laser power, the literature for the analysis of lipids in food has a considerably ample range, from 10%^30^ to 95%,^45^ as it depends on both the MALDI matrix and the sample. In our case, this parameter was tested in the range of 30% to 80%. Spectra acquired at 30%, showed very low signal intensity and therefore a significantly lower number of signals (see Figure 2. Best conditions for honey in Supporting Information Table 2S and Figure 2S, and for bee pollen in Supporting Information Figure 3S ), so lower percentages of laser power were not applied. At the maximum power (80%), the sample suffered thermal degradation, compromising reproducibility; thus, results at that setting were not included in Table 3, and no higher power was tested. Although intensities at 50% and 60% laser power were comparable, 50% yielded a higher number of identifiable lipid peaks. Notably, some of the additional peaks observed at 60% appeared at higher *m*/*z* values but could not be confidently assigned to any lipid species in the LIPID MAPS database^29^ within the accepted mass error threshold, and therefore, these were excluded from analysis. Both the previous and the subtraction of MALDI matrix peaks that could be more intense due to the laser power being higher might be the causes of a slightly higher number of signals at 50% laser power.

**Figure 2:**
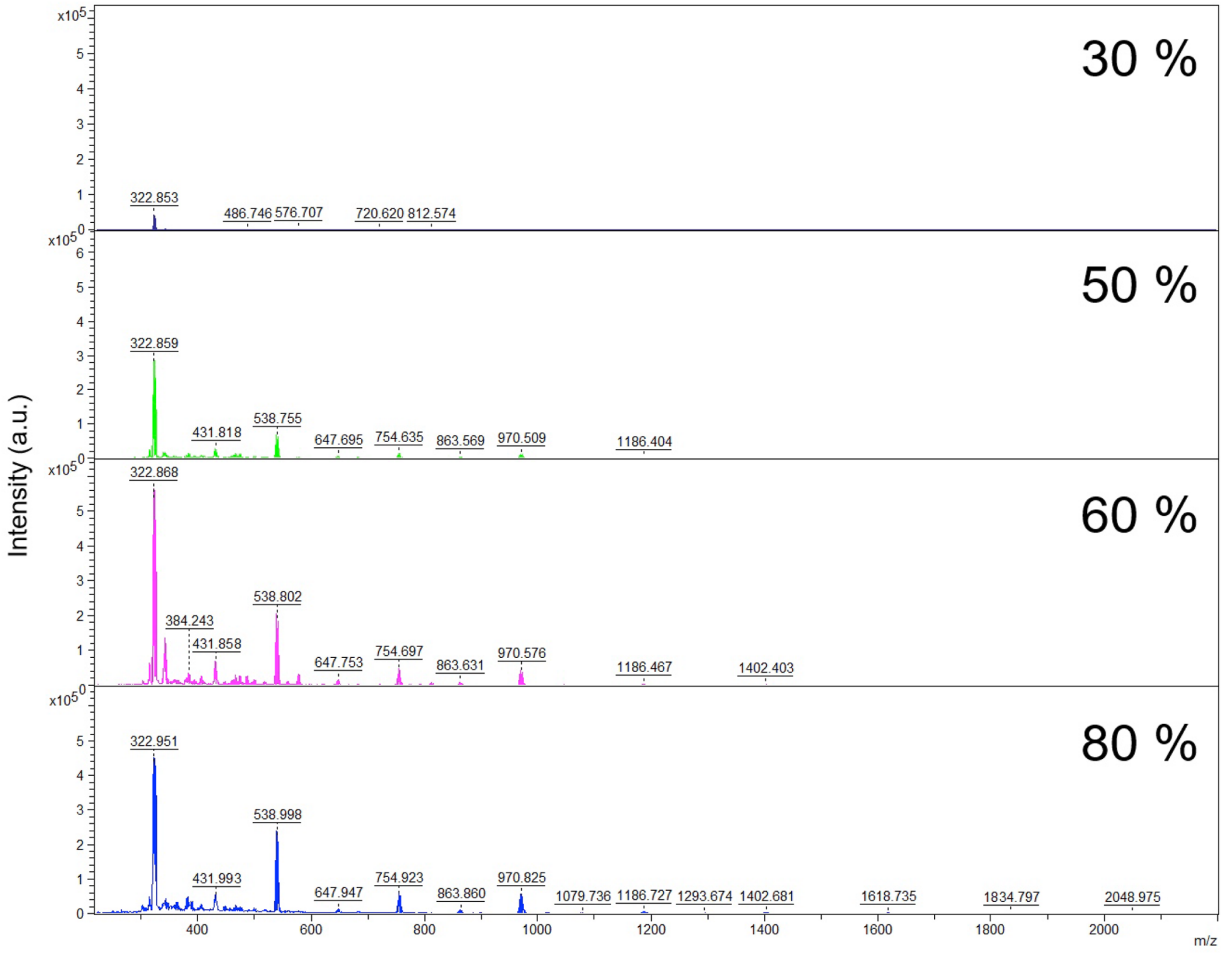
Comparative Mass Spectra of Honey Samples Acquired at 30%, 50%, 60%, and 80% Laser Intensity in MALDI-TOF MS.

The ratio between sample, MALDI matrix, and ionizing agent also affected the number of lipids detected. Excessive proportion of AgTFA led to a dominance of MALDI matrix-derived peaks and a decrease in lipid-identifiable signals, or in some cases, the appearance of nonattributable peaks. After testing ratios from 1:1:0.25 to 1:1:1, the optimal composition was determined to be 1:1:0.25 for honey samples and 1:1:0.5 for bee pollen samples (see Table 3).

### 3.3 Optimization of the Sample Treatment Using Design of Experiments

Next step was focused on optimizing sample preparation parameters, specifically sample amount and extraction time. For this, a quadratic model using central composite face-centered (CCF) design was implemented, aiming to maximize the response variable, which was defined as the number of lipid signals detected after database matching with LIPID MAPS,^29^ and also after subtraction of MALDI matrix-related peaks, as described previously. The factor levels were coded as high (+1), central point (0) and low (−1) levels. For honey, the sample weight factor varied between 250 and 750 mg, while for bee pollen it ranged from 10 to 100 mg. These values agreed with previous published articles, which are mostly lower than 1 g (see Supporting Information Table 1S), and are also related to the amounts of sample availability. Extraction time was evaluated for each of three extraction methods; ultrasonic bath and Vibromatic shaker times were tested from 5 to 15 min, whereas vortex went between 30 and 90 s. The software generated a total of 11 experimental runs for each extraction device, resulting in 33 trials per sample type (honey and bee pollen, respectively).

For honey samples, after fitting individual models for each extraction technique, the Vibromatic shaker yielded the highest number of lipid-associated signals under optimal conditions, with a total of 52 lipids detected. Therefore, this method was selected for further discussion. The best model fitting was obtained by transforming the response using the negative logarithmic function, NegLog (−10 × log (100 – *Y*)), where *Y* represents the number of lipid signals. Significant factors in the model included sample amount, extraction time, and two of their interaction terms: amount with itself and with time. The model showed good statistical parameters, such as a *R* ^2^ of 0.976, *Q* ^2^ of 0.849 (classified as good model by MODDE), model validity of 0.936, reproducibility of 0.905, and a relative standard deviation (RSD) of 0.010. The response contour plot (see Figure 3A) indicated that optimal conditions were achieved using 750 mg of honey and 5 min of agitation using the Vibromatic shaker.

**Figure 3:**
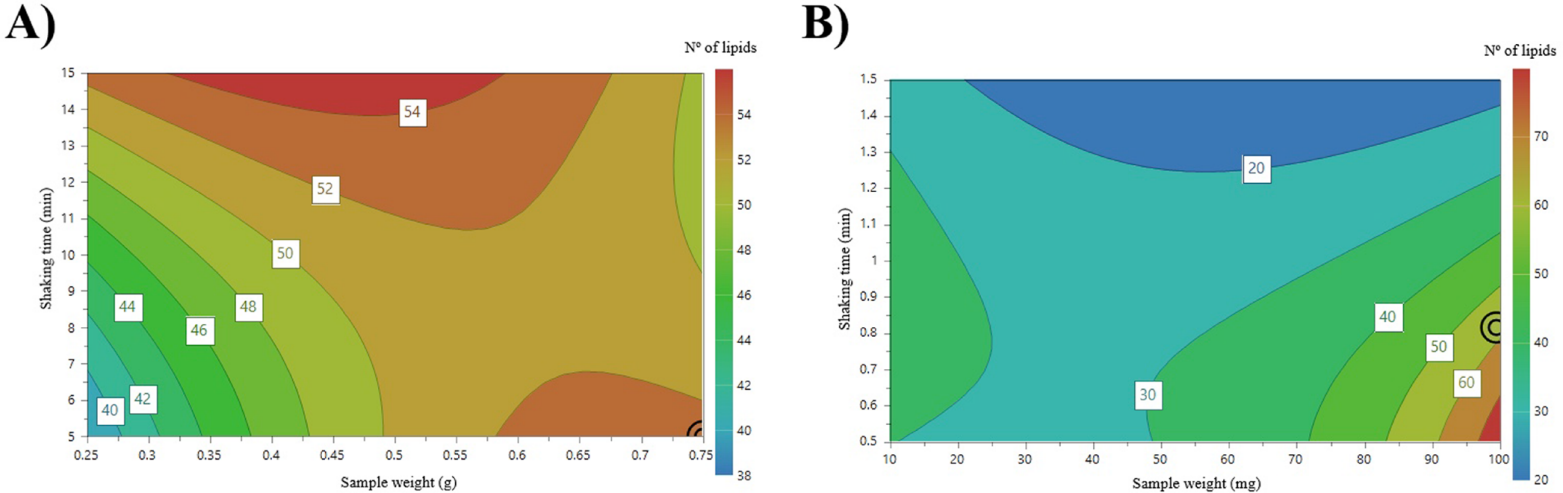
Response Contour Plots From the Design of Experiments for Sample Weight and Shaking Time During Sample Preparation for A) Honey and B) Bee Pollen.

For bee pollen samples, after fitting and optimizing the models for each extraction device, the vortex mixer provided the highest number of lipid-associated signals under optimal conditions, this being 57 signals. Consequently, this method was selected for discussion in this case. The best model performance was achieved by transforming the response using the logarithmic function Log (10 × log (*Y*)), where, again, *Y* corresponds to the number of lipid signals. Significant factors included extraction time, and the same interaction terms as before. For this MALDI matrix, *R* ^2^ was of 0.979, *Q* ^2^ of 0.878 (good model), model validity of 0.970, reproducibility of 0.857, and RSD of 0.034. The contour plot of the response surface (see Figure 3B) shows the optimal conditions which were 99 mg of bee pollen and 48 s of vortex mixing. It must be mentioned that although problematic experimental runs in terms of statistical results were repeated, three data points still had to be excluded from the dataset to ensure proper model fitting and predictive reliability for this MALDI matrix.

### 3.4 Application of the Method

The optimized method was applied to a total of 15 honey and 13 bee pollen samples of diverse botanical and geographical origins (see Table 1). For each sample, signals from spectra were sent to LIPID MAPS,^29^ and the MALDI matrix signals were removed. Then, using the list generated by the database, a construction of a global lipid profile table was carried out for each kind of sample. In instances where a single plausible option remained, that lipid was reported. When multiple candidate identifications were obtained, we considered not only the closest mass match but also the chemical plausibility of the adduct. For example, Ag^+^ adducts were retained given their direct connection to the ionizing agent employed. Confidence in these assignments was further strengthened when both ^107^Ag and ^109^Ag adducts were observed for the same lipid species. The compiled data were then employed to carry out a PCA for honey and bee pollen in terms of botanical and geographical origin.

Complete tables linking each code to its corresponding lipid are provided in the Supporting Information Tables 3S and 4S. Lipid species are referred to using the terms HL (honey lipid) and PL (bee pollen lipid), respectively, followed by the numerical code assigned during statistical analysis. It should be noted that the same lipid may have different codes in honey and bee pollen datasets, depending on their order within each sample.

#### 3.4.1 Honey

A total of 375 distinct lipid species were detected across the 15 honey samples analyzed (H1 to H15), and the PCA reduced the variable set to 12 principal components that together explained 94% of the variance. Among the most relevant lipids contributing to this PCA were ST 30:6;O7, FA 28:7, FAHFA 33:3;O, LPE 14:1, LPE 17:2, LPG 18:3, LPG O-16:0, MIPC 44:0;O2, PG O-38:3, PI 43:6, PS O-41:1, ST 24:0;O4 and ST 26:0;O5. Using these components, the analysis allowed for classification of all honey samples according to their botanical origin (see Supporting Information Table 5S). The average values of the first two principal components are represented in Figure 4A, where clear separation between botanical groups is observed. Regarding geographical origin, differentiation was also seen among the three identified regions, as illustrated in Figure 4B. Samples labeled as “Spain” correspond to those not falling within the other two specific regions. Again, 100% classification accuracy was achieved (see Supporting Information Table 6S), confirming the method’s strong potential to differentiate honeys based on both botanical and geographical origin through their lipidomic profiles.

**Figure 4:**
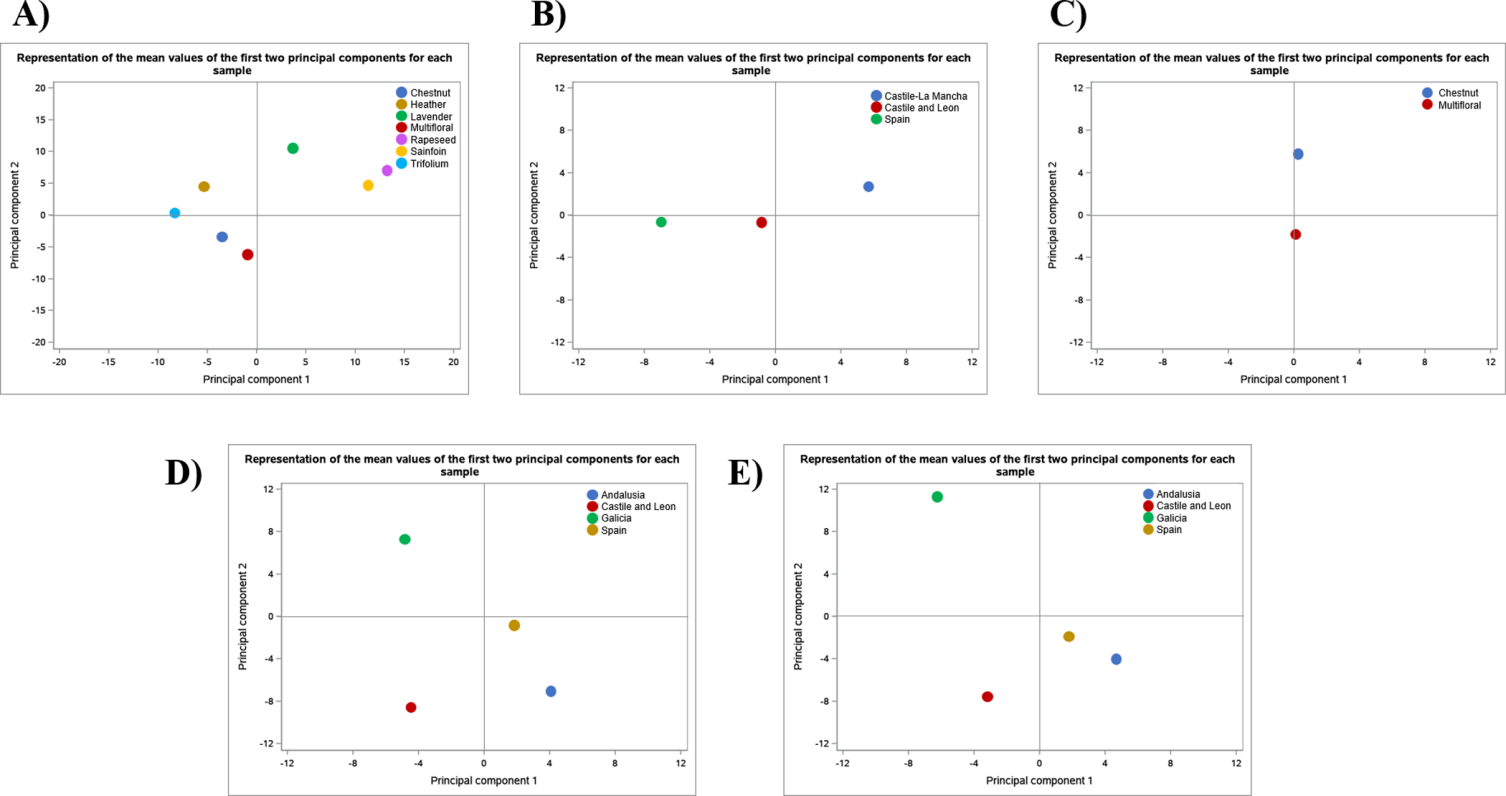
Principal Component Analysis Score Plots Based on Mean Values for: A) Botanical Origin in Honey, B) Geographical Origin in Honey, C) Botanical Origin in Bee Pollen, D) Geographical Origin in Bee Pollen, E) Geographical Origin Within the Same Botanical Origin in Multifloral Bee Pollen.

To better visualize the lipid distribution, Figure 5A presents the relative proportion of each major lipid class in a color-coded graph. This shows that all lipid families were detected across the honey samples except in H5, where no sphingolipids were observed. Overall, glycerophospholipids were the most abundant family (37%), followed by sterol lipids (24%) and then sphingolipids (19%), fatty acyls (11%) and glycerolipids (9%). However, it is important to note that this observation is based on the variety of lipid species detected, as no quantitative analysis was performed. It is also worth noting that certain lipids were particularly recurrent across the honey samples. For instance, lipids FA 36:0 and WE 36:0 were found in all 15 samples. Lipids PC 41:0 and PE 44:0 appeared in 13 out of 15, both missing from H1. Additionally, a group of lipids (ACer 50:1;O2, DG 44:2, MIPC 36:0;O2, PC 44:1, PC O-42:1, and ST 21:2;O3;S) were consistently absent in the same three samples (H5, H6, and H7).

**Figure 5:**
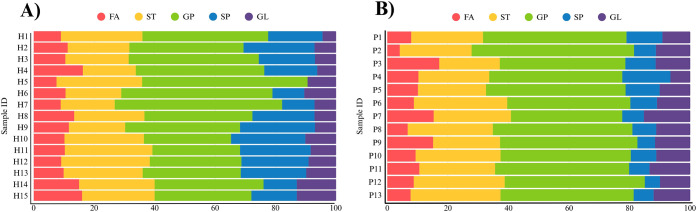
Distribution of lipids across the five main lipid families according to LIPID MAPS^26^ in A) honey and B) bee pollen (FA, fatty acyls; ST, sterol lipids; GP, glycerophospholipids: SP, sphingolipids; GL, glycerolipids).

#### 3.4.2 Bee Pollen

In this case, a total of 337 lipid species were identified across the 13 bee pollen samples (P1 to P13). The PCA reduced the dataset to 10 principal components and these together accounted for 92% of the variance. The most relevant lipids contributing to this model were CE 22:4, FA 42:0;O, LPE 18:3, PA 40:0, PA O-40:2, PG 33:0, PI O-38:1, ST 28:0;O5. Based on this PCA, 11 out of the 13 samples were classified according to their botanical origin (see Table 7S, Supporting Information). Figure 4C illustrates the average values for each group, showing clear differentiation among them. For geographical origin, the samples exhibited a clear separation between the four regions considered. This is shown in Figure 4D, where 100% classification accuracy was achieved (see Table 8S, Supporting Information). Unlike with honey, there were more multifloral samples available, and so, an additional analysis was carried out to assess whether a geographical differentiation could be achieved within the same botanical origin. As shown in Table 9S (Supporting Information), the classification by geographical origin was successful. Figure 4E displays the average PCA values, illustrating a good separation between geographical origins within this botanical group.

Figure 5B illustrates the distribution of lipid classes per bee pollen sample in the same way it was considered for honey, showing that all lipid families were detected across the samples. Overall, glycerophospholipids were the most abundant family (44%), followed by sterol lipids (26%), and then glycerolipids (11%), fatty acyls (10%) and sphingolipids (9%) in proportions quite similar to honey as well. Lipids PC 42:3, PC 42:4, PC 44:3, PS O-42:0, ST 19:2;O3, and TG 43:0 were present in all 13 samples. Lipids Cer 44:0;O4 and HexCer 38:0;O2 appeared in 12 of them, missing only in sample P5, while DG 44:2 was detected in 11 samples, absent in P2 and P9.

#### 3.4.3 Merits of the Proposed Method

The present study is highly novel, as up-to-date only one publication^27^ reported the use of MALDI-TOF MS to analyze the lipid profile in bee pollen samples. Moreover, lipids in honey have never been determined by this technique and only a few works have taken this family of analytes into account at all.^6,46,47^ Some early works indicated probable presence of TGs, STs, GPs^47^ or cholesterol esters (CEs)^46^ in honey samples. Similarly, we have found lipid signals related to all of the previously mentioned families (see Supporting Information Table 3S). The lipidic profiles reported in our article show promising results as they indicate that these bioactive compounds might be relevant to distinguishing between samples from different botanical and geographical origins. In order to obtain these tentative lipidomes, sample preparation and detection conditions were exhaustively optimized, including a design of experiments. With our work, we contribute additional data to the scientific literature and set the foundations for further research in constituents in food matrices with potential for biomarker applications. However, further research is needed to expand the analysis to a broader range of samples from different botanical families and geographical regions.

## 4 Green and Blue Assessment

In the field of analytical chemistry, recent efforts have focused on developing methodologies that align with the principles of white analytical chemistry (WAC).^48^ This multidimensional approach seeks not only analytical efficiency (red, R), but also environmental sustainability (green, G) and practical applicability (blue, B).^49^ Since the early 2020s, a wide variety of tools, metrics, and templates have been introduced in this context, aimed not only at assessing the RGB dimensions but also at evaluating the innovativeness of analytical methods,^50^ and even at visually presenting their main features and results.^51^ For the red dimension, the red analytical performance index (RAPI) was proposed to evaluate analytical performance, including key validation parameters.^52^ However, as the proposed MALDI-TOF method was not designed or optimized for quantitative analysis, but rather for exploratory lipid profiling and comparative purposes, this dimension was not considered. As in previous studies,^28^ both the green and blue dimensions were thoroughly assessed using the analytical GREEnness calculator (AGREE),^53^ the modified green analytical procedure index (MoGAPI),^54^ and the blue applicability grade index (BAGI),^55^ respectively (see Supporting Information, Figure 4S). All three tools are freely available with user-friendly software and implementation guidelines.

AGREE offers a comprehensive sustainability assessment based on the 12 principles of green analytical chemistry,^56^ transformed into a 0–1 scoring scale. The final score reflects the combined contribution of all principles, and results are visualized as a circular pictogram using a red-yellow-green color gradient. A method is generally considered “green” when the score exceeds 0.6. MoGAPI provides a more detailed evaluation of method greenness by incorporating a broader range of parameters with stricter and more specific criteria. Results are displayed in a pentagonal chart using the same color-based system.^57–74^

As displayed in Figure 4SA and Figure 4SB, the AGREE scores differed slightly between honey and bee pollen according to their sample preparation workflows (see Figure 1). Offline measurements (principle 3), and the high energy consumption of MALDI-TOF MS (principle 9) were negatively weighted by the AGREE metric (see Figure 4SA). In contrast, the minimal sample size (principle 2), a fast sample preparation process involving just a few steps (extraction and centrifugation, principle 4) without derivatization (principle 6), the possibility of simultaneous detection of a high number of analytes and samples per hour (principle 8), and the operator safety (principle 12) were highlighted in green. It is important to note that, although the energy consumption of the instrument is high compared to others, the use of MALDI-TOF MS is the best option for tentative lipidic profiling. The final AGREE scores were 0.66 for honey and 0.68 for bee pollen, both above the optimal threshold (0.6), indicating that the proposed analytical methods were environmentally friendly. Regarding MoGAPI (see Figure 4SC), the score obtained was 70 out of 100, which corresponded to a moderate environmental impact. The results obtained agreed with the previous tool. Some benefits highlighted in green included easy and fast sample preparation, low sample and solvent amounts. By contrast, the main drawbacks stem from the use of nongreen organic solvents (isopropanol, hexane, and dichloromethane), the off-line collection and the energy consumption.

The applicability of an analytical method was evaluated by the blue applicability grade index (BAGI).^55^ This metric based on ten attributes generates a pictogram that reflects the practicality. A sequential blue color scale ranging from dark blue (high compliance) to white (no compliance), is employed to visually represent the final score. According to BAGI guidelines, a final score exceeding 60.0 points is recommended for a method to be considered practical. The proposed method achieved a score of 75. One of the penalties was the use of MALDI-TOF instrumentation, which is not widely available in all laboratories. Similarly, partial automation of the procedure also lowered the score, as some steps were performed manually. However, the use of MALDI-TOF provided fast and accurate analysis for a high number of samples and the simplicity and low cost of the sample preparation and extraction steps, as well as the small sample amount required, contributed positively to the final score. For the first time, three metrics were applied to a MALDI-TOF MS lipidomic study for apicultural products, and they categorized the method as green (environmentally friendly) and blue (practical).

## Supplementary Material



## Data Availability

The datasets generated during the current study are included in this published article, or they are available from the corresponding author upon reasonable request.

## Notes

Additional experimental details including chromatograms, PCA data tables, lipid codes, and green and blue metrics used in this work (PDF)

## Abbreviations Used

AANS: 1-amino-2-naphthol-4-sulfonic acid
AGREE: analytical GREEnness calculator
AgTFA: silver trifluoroacetate
BAGI: blue applicability grade index
CC: cinnamic chloride
CCF: central composite face-centered
CE: cholesterol ester
DCTB: trans-2-[3-(4-*tert*-butylphenyl)-2-methyl-2-propenylidene]­malononitrile
DHAP: 3′,5′-dimethoxy-4-hydroxyacetophenone
DHB: 2,5-dihydroxybenzoic acid
DIT: dithranol
DoE: design of experiments
GAC: green analytical chemistry
GC-FID: gas chromatography coupled with flame ionization detection
H: honey
HL: honey lipid
HMCA: 4-hydroxy-3-methoxycinnamaldehyde
HX: hexane
IPA: isopropanol
LIPID MAPS: lipid metabolites and pathways strateg
LMSD: LIPID MAPS structure database
MoGAPI: modified green analytical procedure index
NaTFA: sodium trifluoroacetate
P: bee pollen
PCA: principal component analysis
PEG: polyethylene glycol
PL: bee pollen lipid
RAPI: red analytical performance index
RSD: relative standard deviation
THF: tetrahydrofuran
UHPLC: ultrahigh-performance liquid chromatography
WAC: white analytical chemistry

## References

[1] Ares A. M., Valverde S., Bernal J. L., Nozal M. J., Bernal J. (2018). Extraction
and Determination of Bioactive Compounds from Bee Pollen. J. Pharm. Biomed. Anal..

[2] Fuente-Ballesteros A., Augé C., Bernal J., Ares A. M. (2023). Development
and
Validation of a Gas Chromatography-Mass Spectrometry Method for Determining
Acaricides in Bee Pollen. Molecules.

[3] Magdas T. M., David M., Hategan A. R., Filip G. A., Magdas D. A. (2024). Geographical
Origin Authentication—A Mandatory Step in the Efficient Involvement
of Honey in Medical Treatment. Foods.

[4] Ratiu I. A., Al-Suod H., Bukowska M., Ligor M., Buszewski B. (2020). Correlation
Study of Honey Regarding Their Physicochemical Properties and Sugars
and Cyclitols Content. Molecules.

[5] Ligor M., Bukowska M., Ratiu I. A., Gadzała-Kopciuch R., Buszewski B. (2020). Determination
of Neonicotinoids in Honey Samples Originated
from Poland and Other World Countries. Molecules.

[6] Valverde S., Ares A. M., Elmore J. S., Bernal J. (2022). Recent Trends in the
Analysis of Honey Constituents. Food Chem..

[7] Chica M. (2012). Authentication
of Bee Pollen Grains in Bright-Field Microscopy by Combining One-Class
Classification Techniques and Image Processing. Microsc. Res. Technol..

[8] Guelpa A., Marini F., du Plessis A., Slabbert R., Manley M. (2017). Verification
of Authenticity and Fraud Detection in South African Honey Using NIR
Spectroscopy. Food Control..

[9] Egido C., Saurina J., Sentellas S., Núñez O. (2024). Honey Fraud
Detection Based on Sugar Syrup Adulterations by HPLC-UV Fingerprinting
and Chemometrics. Food Chem..

[10] Liu X., Chen L., Shen A., Song J., Wang M., Qi X., Zhao S., Hu L. (2025). Advances on Sample Pretreatment and
Identification Tools of Biomarker Analysis for Food Authenticity. Food Control..

[11] Machado
De-Melo A. A., de Almeida-Muradian L.
B., Sancho M. T., Pascual-Maté A. (2018). Composition and Properties of Apis Mellifera Honey:
A Review. J. Apic. Res..

[12] Fuente-Ballesteros A., Priovolos I., Ares A. M., Samanidou V., Bernal J. (2023). Green Sample Preparation
Methods for the Analysis of
Bioactive Compounds in Bee Products: A Review. Adv. Sample Prep..

[13] Martín-Gómez B., Salahange L., Tapia J. A., Martín M. T., Ares A. M., Bernal J. (2022). Fast Chromatographic Determination
of Free Amino Acids in Bee Pollen. Foods.

[14] Ares A. M., Tapia J. A., González-Porto A. V., Higes M., Martín-Hernández R., Bernal J. (2022). Glucosinolates as Markers
of the Origin and Harvesting Period for Discrimination of Bee Pollen
by UPLC-MS/MS. Foods.

[15] Zhou L., Ma Y., Xu J., Hu Y., Zhao M., Marchioni E., Fu H. (2025). Determination and Comparison
of Lipid Profiles of Chinese Green Tea
Varieties Using Untargeted Lipidomics Analysis Combined with Chemometrics. Food Chem..

[16] Yao J., Zhou L., Hu Y., Zhao M., Ma Y., Liu J., Marchioni E. (2023). Combining
Untargeted Lipidomics Analysis and Chemometrics
to Identify the Edible and Poisonous Mushrooms (Pleurotus Cornucopiae
vs Omphalotus Japonicus). J. Agric. Food Chem..

[17] Shi C., Guo H., Wu T., Tao N., Wang X., Zhong J. (2019). Effect of
Three Types of Thermal Processing Methods on the Lipidomics Profile
of Tilapia Fillets by UPLC-Q-Extractive Orbitrap Mass Spectrometry. Food Chem..

[18] Zhu J., Zhou L., Zhao M., Wei F., Fu H., Marchioni E. (2023). Revealing
the Dynamic Changes of Lipids in Coffee Beans
during Roasting Based on UHPLC-QE-HR-AM/MS/MS. Food Res. Int..

[19] Jarukas L., Kuraite G., Baranauskaite J., Marksa M., Bezruk I., Ivanauskas L. (2021). Optimization and Validation of the GC/FID Method for
the Quantification of Fatty Acids in Bee Products. Appl. Sci..

[20] Błońska D., Buszewski B. (2025). Characterization
of Honey Microbiome Using MALDI-TOF
Mass Spectrometry and Physicochemical Study. Molecules.

[21] Engel K. M., Prabutzki P., Leopold J., Nimptsch A., Lemmnitzer K., Vos D. R. N., Hopf C., Schiller J. (2022). A New Update of MALDI-TOF
Mass Spectrometry in Lipid Research. Prog. Lipid
Res..

[22] Walczak J., Pomastowski P., Bocian S., Buszewski B. (2016). Determination
of Phospholipids in Milk Using a New Phosphodiester Stationary Phase
by Liquid Chromatography-Matrix Assisted Desorption Ionization Mass
Spectrometry. J. Chromatogr. A.

[23] Lay J. O., Liyanage R., Durham B., Brooks J. (2006). Rapid Characterization
of Edible Oils by Direct Matrix-Assisted Laser Desorption/Ionization
Time-of-Flight Mass Spectrometry Analysis Using Triacylglycerols. Rapid Commun. Mass Spectrom..

[24] Zhang Y.-X., Zhao X.-B., Ha W., Zhang Y.-D., Shi Y.-P. (2021). Spatial
Distribution Analysis of Phospholipids in Rice by Matrix-Assisted
Laser Desorption/Ionization Time-of-Flight Mass Spectrometry Imaging. J. Chromatogr. A.

[25] Zhang Y.-X., Zhang Y.-D., Shi Y.-P. (2023). Tracking Spatial Distribution Alterations
of Multiple Endogenous Molecules during Lentil Germination by MALDI
Mass Spectrometry Imaging. J. Agric. Food Chem..

[26] Liebisch G., Fahy E., Aoki J., Dennis E. A., Durand T., Ejsing C. S., Fedorova M., Feussner I., Griffiths W. J., Köfeler H., Merrill A. H., Murphy R. C., O’Donnell V. B., Oskolkova O., Subramaniam S., Wakelam M. J. O., Spener F. (2020). Update on
LIPID MAPS Classification, Nomenclature, and Shorthand Notation for
MS-Derived Lipid Structures. J. Lipid Res..

[27] Braglia C., Alberoni D., Di Gioia D., Giacomelli A., Bocquet M., Bulet P. (2024). Application of a Robust MALDI Mass
Spectrometry Approach for Bee Pollen Investigation. Anal. Bioanal. Chem..

[28] Fuente-Ballesteros A., Tian L., Liu L., Ares A. M., Bayen S., Bernal J. (2025). Development and Validation
of a Green and Practical
Method for Studying Pesticides and Related Chemical Compounds in Bee
Pollen Samples by UAE-LC-QTOF-MS. J. Food Compos.
Anal..

[29] Sud M., Fahy E., Cotter D., Brown A., Dennis E. A., Glass C. K., Merrill A. H., Murphy R. C., Raetz C. R. H., Russell D. W. (2007). LMSD:
LIPID MAPS Structure Database. Nucleic Acids
Res..

[30] Schiller J., Süß R., Petković M., Arnold K. (2002). Triacylglycerol Analysis
of Vegetable Oils by Matrix-Assisted Laser Desorption and Ionization
Time-of-Flight (MALDI-TOF) Mass Spectrometry and 31P NMR Spectroscopy. J. Food Lipids.

[31] Zhang Y.-X., Zhang Y.-D., Shi Y. P. (2023). A Reliable
and Effective Sample Preparation
Protocol of MALDI-TOF-MSI for Lipids Imaging Analysis in Hard and
Dry Cereals. Food Chem..

[32] Dannenberger D., Süß R., Teuber K., Fuchs B., Nuernberg K., Schiller J. (2010). The Intact Muscle Lipid Composition of Bulls: An Investigation
by MALDI-TOF MS and 31P NMR. Chem. Phys. Lipids.

[33] Tzompa-Sosa D. A., Dewettinck K., Provijn P., Brouwers J. F., de Meulenaer B., Oonincx D. G. A. B. (2021). Lipidome of Cricket Species Used as Food. Food Chem..

[34] Krejčí P., Žingor Z., Balarynová J., Čevelová A., Tesárek M., Smýkal P., Bednář P. (2025). Modern Comprehensive
Metabolomic Profiling of Pollen Using Various Analytical Techniques. Molecules.

[35] Zhou M., Li T., Shen L., Zhong Q., Huang T., Zhou T. (2025). Enhanced Authentication
of Organic Milk Using MALDI-TOF MS with Combined Lipid-Peptide Fingerprinting
and Machine Learning Integration. Food Chem..

[36] Lau W. C. D., Donnellan L., Harris J. C., Seidel J., Hayes J. E., Croser J., Hoffmann P. (2025). Coupling Proteomics and Lipidomics
for Insights into Regulation of Oat (Avena Sativa) Grain Lipid Synthesis. Food Chem..

[37] Fernandes C., Figueira E., Tauler R., Bedia C. (2018). Exposure to Chlorpyrifos
Induces Morphometric, Biochemical and Lipidomic Alterations in Green
Beans (Phaseolus Vulgaris). Ecotoxicol. Environ.
Saf..

[38] Zhang H., Olson D. J. H., Van D., Purves R. W., Smith M. A. (2012). Rapid Identification
of Triacylglycerol-Estolides in Plant and Fungal Oils. Ind. Crops Prod..

[39] Cheema S. K., Grimwade-Mann M., Weaver G., Collins B., Shenker N., Cameron S. (2025). Freeze-Drying
Donor Human Milk Allows Compositional
Stability for 12 Months at Ambient Temperatures. J. Food Compos. Anal..

[40] Leopold J., Popkova Y., Engel K. M., Schiller J. (2018). Recent Developments
of Useful MALDI Matrices for the Mass Spectrometric Characterization
of Lipids. Biomolecules.

[41] Sacchi R., Cutignano A., Picariello G., Paduano A., Genovese A., Siano F., Nuzzo G., Caira S., Lubritto C., Ricci P. (2020). Olive Oil from the 79 A.D. Vesuvius Eruption Stored
at the Naples National Archaeological Museum (Italy). Npj Sci. Food.

[42] Picariello G., Sacchi R., Addeo F. (2007). One-Step Characterization
of Triacylglycerols
from Animal Fat by MALDI-TOF MS. Eur. J. Lipid
Sci. Technol..

[43] Peršurić Ž., Saftić Martinović L., Zengin G., Šarolić M., Kraljević Pavelić S. (2020). Characterization of Phenolic and
Triacylglycerol Compounds in the Olive Oil By-Product Pâté
and Assay of Its Antioxidant and Enzyme Inhibition Activity. lwt -Food Sci. Technol..

[44] Choi S.-S., Ha S.-H. (2008). Influence of Sample Preparation Method and Silver Salt Types on MALDI-TOFMS
Analysis of Polybutadiene. Macromol. Res..

[45] England P., Tang W., Kostrzewa M., Shahrezaei V., Larrouy-Maumus G. (2020). Discrimination of Bovine Milk from
Non-Dairy Milk by
Lipids Fingerprinting Using Routine Matrix-Assisted Laser Desorption
Ionization Mass Spectrometry. Sci. Rep..

[46] Kapoulas V. M., Mastronicolis S. K., Galanos D. S. (1977). Identification of the Lipid Components
of Honey. Z. Lebensm. -Unters. Forsch..

[47] Smith M. R., McCaughey W. F. (1966). Identification
of Some Trace Lipids in Honey. J. Food Sci..

[48] Nowak P. M., Wietecha-Posłuszny R., Pawliszyn J. (2021). White Analytical
Chemistry: An Approach to Reconcile the Principles of Green Analytical
Chemistry and Functionality. TrAC, Trends Anal.
Chem..

[49] Fuente-Ballesteros A., Samanidou V., Ares A. M., Bernal J. (2025). Ten Principles for
Developing and Implementing Tools in the Context of White Analytical
Chemistry. Sustainable Chem. Pharm..

[50] Fuente-Ballesteros A., Martínez-Martínez V., Ares A. M., Valverde S., Samanidou V., Bernal J. (2025). Violet Innovation Grade Index (VIGI):
A New Survey-Based Metric for Evaluating Innovation in Analytical
Methods. Anal. Chem..

[51] Fuente-Ballesteros A., Jano A., Ares A. M., Valverde S., Bernal J. (2025). GLANCE: A
Novel Graphical Tool for Simplifying Analytical Chemistry Method Evaluation. Analytica.

[52] Nowak P. M., Wojnowski W., Manousi N., Samanidou V., Płotka-Wasylka J. (2025). Red Analytical
Performance Index (RAPI) and Software:
The Missing Tool for Assessing Methods in Terms of Analytical Performance. Green Chem..

[53] Pena-Pereira F., Wojnowski W., Tobiszewski M. (2020). AGREE - Analytical GREEnness Metric
Approach and Software. Anal. Chem..

[54] Mansour F. R., Płotka-Wasylka J., Locatelli M. (2024). Modified GAPI (MoGAPI) Tool and Software
for the Assessment of Method Greenness: Case Studies and Applications. Analytica.

[55] Manousi N., Wojnowski W., Płotka-Wasylka J., Samanidou V. (2023). Blue Applicability
Grade Index (BAGI) and Software: A New Tool for the Evaluation of
Method Practicality. Green Chem..

[56] Gałuszka A., Migaszewski Z., Namieśnik J. (2013). The 12 Principles of Green Analytical
Chemistry and the SIGNIFICANCE Mnemonic of Green Analytical Practices. TrAC, Trends Anal. Chem..

[57] Calvano C. D., Zambonin C. G., Foti C., Cassano N., Vena G. A. (2008). A Matrix
Assisted Laser Desorption Ionization Time-of-Flight Mass Spectrometry
Investigation to Assess the Composition of Cod Liver Oil Based Products
Which Displayed a Different in Vivo Allergenic Power. Food Chem. Toxicol..

[58] Adel A., El-Baz A., Shetaia Y., Sorour N. M. (2021). Biosynthesis of
Polyunsaturated Fatty Acids by Two Newly Cold-Adapted Egyptian Marine
Yeast. 3 Biotech..

[59] Ayorinde F. O., Garvin K., Saeed K. (2000). Determination
of the Fatty Acid Composition
of Saponified Vegetable Oils Using Matrix-Assisted Laser Desorption/Ionization
Time-of-Flight Mass Spectrometry. Rapid Commun.
Mass Spectrom..

[60] Bianco M., Ventura G., Coniglio D., Monopoli A., Losito I., Cataldi T. R. I., Calvano C. D. (2024). Development
of a New Binary Matrix
for the Comprehensive Analysis of Lipids and Pigments in Micro- and
Macroalgae Using MALDI-ToF/ToF Mass Spectrometry. Int. J. Mol. Sci..

[61] Floriano
Oreano de Azevedo P., Aranha Martins M., Blank M., Cardoso L., de Aguiar Bertaglia E., Rabelo Lisboa T., Tomas Jerônimo G., Furtado W. E., Laterça Martins M. (2021). Dietary Supplementation
of Levamisole Modulates Protein and Lipid MALDI-TOF MS Profiles of
Nile Tilapia without Causing Negative Histological Alterations. Aquaculture.

[62] Bode L., Beermann C., Mank M., Kohn G., Boehm G. (2004). Human and
Bovine Milk Gangliosides Differ in Their Fatty Acid Composition. J. Nutr..

[63] Fuchs B., Bischoff A., Süß R., Teuber K., Schürenberg M., Suckau D., Schiller J. (2009). Phosphatidylcholines
and -Ethanolamines
Can Be Easily Mistaken in Phospholipid Mixtures: A Negative Ion MALDI-TOF
MS Study with 9-Aminoacridine as Matrix and Egg Yolk as Selected Example. Anal. Bioanal. Chem..

[64] Alves E., Melo T., Rey F., Moreira A. S. P., Domingues P., Domingues M. R. (2016). Polar Lipid
Profiling of Olive Oils as a Useful Tool
in Helping to Decipher Their Unique Fingerprint. lwt -Food Sci. Technol..

[65] Li B., Stuart D. D., Shanta P. V., Pike C. D., Cheng Q. (2022). Probing Herbicide
Toxicity to Algae (Selenastrum Capricornutum) by Lipid Profiling with
Machine Learning and Microchip/MALDI-TOF Mass Spectrometry. Chem. Res. Toxicol..

[66] Li S., Ng T.-T., Yao Z.-P. (2021). Quantitative
Analysis of Blended
Oils by Matrix-Assisted Laser Desorption/Ionization Mass Spectrometry
and Partial Least Squares Regression. Food Chem..

[67] Piras C., Ceniti C., Hartmane E., Costanzo N., Morittu V. M., Roncada P., Britti D., Cramer R. (2020). Rapid Liquid AP-MALDI
MS Profiling of Lipids and Proteins from Goat and Sheep Milk for Speciation
and Colostrum Analysis. Proteomes.

[68] Cutignano A., Siano F., Romano R., Aiello A., Pizzolongo F., Berni Canani R., Paparo L., Nocerino R., Di Scala C., Addeo F., Picariello G. (2020). Short-Term Effects of Dietary Bovine
Milk on Fatty Acid Composition of Human Milk: A Preliminary Multi-Analytical
Study. J. Chromatogr. B: anal. Technol. Biomed.
Life Sci..

[69] Teuber K., Schiller J., Fuchs B., Karas M., Jaskolla T. W. (2010). Significant
Sensitivity Improvements by Matrix Optimization: A MALDI-TOF Mass
Spectrometric Study of Lipids from Hen Egg Yolk. Chem. Phys. Lipids.

[70] Dyer J. M., Deb-Choudhury S., Cornellison C. D., Krsinic G., Dobbie P., Rosenvold K., Clerens S. (2014). Spatial and Temporal Mass Spectrometric
Profiling and Imaging of Lipid Degradation in Bovine M. Longissimus
Dorsi Lumborum. J. Food Compos. Anal..

[71] Vieler A., Wilhelm C., Goss R., Süß R., Schiller J. (2007). The Lipid Composition of the Unicellular Green Alga
Chlamydomonas Reinhardtii and the Diatom Cyclotella Meneghiniana Investigated
by MALDI-TOF MS and TLC. Chem. Phys. Lipids.

[72] Dias
Schleder D., Blank M., Buglione Peruch L. G., Vieira F. D. N., Andreatta E. R., Hayashi L. (2017). Thermal Resistance
of Pacific White Shrimp Fed Sargassum Filipendula: A MALDI-TOF Mass
Spectrometry Approach. Aquaculture.

[73] Shi Q., Zhang X., Liu X., Yan C., Lu S. (2024). Visualization
of PFOA Accumulation and Its Effects on Phospholipid in Zebrafish
Liver by MALDI Imaging. Anal. Bioanal. Chem..

[74] Wang X., Chen Y., Liu Y., Ouyang L., Yao R., Wang Z., Kang Y., Yan L., Huai D., Jiang H., Lei Y., Liao B. (2022). Visualizing the Distribution
of Lipids in Peanut Seeds by MALDI Mass Spectrometric Imaging. Foods.

