# Supplementary material for: MALDI-TOF MS-Based Lipidomic Profile of Honey and Bee Pollen
Source: ACS Agric Sci Technol. 2025 Nov 6;5(12):2585–95. doi: 10.1021/acsagscitech.5c00883 (PMC12710861; doi:10.1021/acsagscitech.5c00883)

SUPPORTING INFORMATION

MALDI-TOF MS-Based Lipidomic Profile of Honey and Bee Pollen

Ana Jano[1], Adrián Fuente-Ballesteros[1], Jesús A. Tapia[1,2], Silvia Valverde[1], Ana M. Ares[1], José Bernal[1*]

[1]Analytical Chemistry Group (TESEA), I. U. CINQUIMA, Faculty of Sciences, University of Valladolid, 47011, Valladolid, Spain.

[2]Department of Statistics and Operations Research, Faculty of Sciences, University of Valladolid, 47011 Valladolid, Spain

***Corresponding author:** Tel# 34-98-318-6347; jose.bernal@uva.es

Table 1S. Overview of Research Articles Using MALDI-TOF for Lipid Analysis in Food Products.

Food Sample	Lipid Family[a]	MALDI Matrix	Extraction Solvent	Sample Amount	Ref.
Cod liver oil	GL	DHB	Pentane	50 mg	57
Rice, wheat	GL, GP	9-AA, DHB	MeOH	200 mg	31
Fish	FA	NS	$CHCl_3$:MeOH (2:1)	230 - 300 mg	58
Olive oil,Pâté	GL	DHB	THF	3 g	43
Oat	GL, GP	CHCA	MTBE:MeOH (3:1) or $CHCl_3$:MeOH (2:1)	50 mg	36
Milk	GP	DHB	$CHCl_3$:MeOH (2:1)	2 ml or 2 mg	22
Vegetable oils	FA	F20TPP	MeOH: H_2O (4:1)	20 mg	59
Micro and macroalgae	FA, GP, GL	DAN & 9-AA	MeOH:$CHCl_3$ (2:1)	200 mg	60
Fish	GP	SA, DHB	NS	NS	61
Bovine and soya milks	GL GP	sDHB	H_2O	1 mL	45
Organic milk	FA, GP	DHB, S-DHB, CHCA	MeOH, MTBE	20 µL	35
Green beans	GP	DHB	MTBE:MeOH (1:1)	5 mg	37
Human milk	NS	DHB, SA	NS	1 µL	39
Human milk	FA	DHB	Diisopropylether:1-butanol (6:4)	10 mL	62
Criket	GL, ST, GL	NS	DCM:MeOH (2:1)	5 g	33
Olive oil	FA	DHB	$CHCl_3$	5 mg	41
Animal fat	GL	DHB	$CHCl_3$	10 mg	42
Hen egg yolk	GP	DHB, 9-AA	$CHCl_3$:MeOH:H_2O (1:1:1)	NS	63
Olive oils	GL	DHB	$CHCl_3$	10 mg	64
Algae	GL	sDHB	NS	NS	65
Vegetable oil	GL	DHB & CHCA	No solvent used	NS	66
Edible oils	GL	DHB	HX	1 mg	23
Plant and fungal oils	GL	THAP	$CHCl_3$	NS	38
Goat and Sheep Milk	FA, GP, SP	CClCA, CHCA	$CHCl_3$:MeOH (1:2), H_2O:$CHCl_3$ (1:1)	50 µL	67
Human milk	FA	DHB	$CHCl_3$	10 mL	68
Hen egg yolk	GP, SP, GL	Di-FCCA, 9-AA	$CHCl_3$:MeOH:H_2O (1:1:1)	NS	69
Bovine meat	GP, FA, GP, GL, ST	CHCA,9-AA	$CHCl_3$:MeOH (2:1)	10 g	70
Rice	GL	DHB	No solvent used	NS	24
Muscle of bulls	GL	DHB, 9-AA	$CHCl_3$:MeOH (2:1)	2 g	32

Algae	GL	DHB	$CHCl_3$:MeOH (2:1)	NS	[71]
White shrimp	GP	DHB	NS	NS	[72]
Lentil	GL, GP	CHCA, 9-AA	No solvent used	NS	[25]
Vegetable oils	GL	DHB	$CHCl_3$	1 mg	[30]
Zebrafsh liver	GP	DAN	No solvent used	NS	[73]
Peanut Seeds	GL, GP	DHB	No solvent used	NS	[74]

[a]Classification according to LIPID MAPS® Database.[26] **9-AA**, 9-aminoacridine; **CClCA**, 4-chloro-α-cyano-cinnamic acid; **CHCA**, α-cyano-4-hydroxycinnamic acid; **$CHCl_3$**, chloroform; **DAN**, 1,5-diaminonaphthalene; **DCM**, dichloromethane; **DHB**, 2,5-dihydroxybenzoic acid; **Di-FCCA**, difluorocinnamic acid; **FA**, fatty acids; **F20TPP**, *meso*-tetrakis(pentafluorophenyl)porphyrin; **GL**, glycerolipids; **GP**, glycerophospholipids; **H_2O**, water; **HX**, hexane; **MeOH**, methanol; **MTBE**, methyl tert-butyl ether; **NS**, not specified; **sDHB**, super-DHB; **SA**, sinapinic acid; **SP**, sphingolipids; **ST**, sterol lipids; **THAP**, 2′,4′,6′-trihydroxyacetophenone; **THF**, tetrahydrofuran.

Table 2S. Identified Lipids in Honey With 50% Laser Intensity Using a Sample:Matrix:Agent Ratio of 1:1:0.25. MALDI Matrix is DHAP and Ionizing Agent is AgTFA in THF (10 mg mL^{-1}).

Input Mass	Matched Mass	Delta	Name
404.187	404.1963	0.0093	SPBP 17:1;O2
406.187	406.1869	0.0001	NAE 16:0
406.187	406.1869	0.0001	SPB 18:1;O2
409.008	409.0563	0.0483	ST 18:4;O4
411.172	411.1754	0.0034	FA 20:5;O4
413.176	413.1782	0.0022	FA 18:3;O7
427.814	427.8171	0.0031	PS O-42:4
429.816	429.8146	0.0014	PS 41:2
431.818	431.8172	0.0008	PC 38:0
431.818	431.8172	0.0008	PE 41:0
433.820	433.8277	0.0077	IPC 40:0;O2
435.821	435.8198	0.0012	PC O-40:3
437.175	437.1725	0.0025	ST 24:6;O5
448.846	448.8588	0.0128	PC 44:3
459.181	459.1863	0.0053	FA 22:3;O
461.179	461.1815	0.0025	FA 21:2;O2
461.179	461.1815	0.0025	MG 18:2
466.786	466.7730	0.013	PS 44:8
468.782	468.7887	0.0067	PS 44:6
470.789	470.8043	0.0153	PS 44:4
517.512	517.5166	0.0046	FA 33:0;O
643.695	643.6575	0.0375	FA 42:0;O
645.695	645.6908	0.0042	FA 45:0
649.697	649.6857	0.0113	WE 44:0
680.668	680.6915	0.0235	Cer 44:0;O2
682.671	682.6684	0.0026	Cer 44:2;O2
684.673	684.6653	0.0077	CE 19:0
750.634	750.6371	0.0031	PA O-39:0
752.633	752.6399	0.0069	HexCer 39:1;O2
754.635	754.6296	0.0054	Cer 44:1;O4
754.635	754.6296	0.0054	PC O-34:0
754.635	754.6296	0.0054	PE O-37:0
756.638	756.6348	0.0032	HexCer 38:1;O2
756.638	756.6348	0.0032	HexCer 38:0;O3
758.640	758.6422	0.0022	CerP 44:1;O2
758.640	758.6422	0.0022	PC O-36:0
758.640	758.6422	0.0022	PE O-39:0
760.648	760.6450	0.003	TG 44:4
812.603	812.6012	0.0018	HexCer 38:0;O3
859.569	859.5695	0.0005	PI 37:2
859.569	859.5695	0.0005	TG 51:14;O2
861.569	861.5664	0.0026	TG 53:15;O2
863.569	863.5644	0.0046	PI 36:2
865.573	865.5702	0.0028	PS 41:7
867.577	867.5858	0.0088	PS 41:6
964.508	964.5032	0.0048	PS 41:3
966.506	966.5136	0.0076	PC 42:7
968.506	968.5134	0.0074	PC 42:7
970.509	970.5201	0.0111	SHexCer 40:1;O2
972.514	972.5199	0.0059	SHexCer 40:1;O2
974.515	974.5034	0.0116	PS 42:4

976.521	976.5190	0.002	PS 42:3

Table 3S. Identified Lipids in Honey Samples and Their Associated Codes.

Code	Lipid	Code	Lipid	Code	Lipid	Code	Lipid	Code	Lipid
**HL1**	ACer 48:1;O2	**HL76**	LPA 17:2	**HL151**	PC 41:1	**HL226**	PS 41:2	**HL301**	ST 27:5;O3
**HL2**	ACer 50:1;O2	**HL77**	LPA 18:0	**HL152**	PC 41:2	**HL227**	PS 44:0	**HL302**	ST 28:0;O
**HL3**	ACer 56:1;O2	**HL78**	LPA 18:3	**HL153**	PC 41:6	**HL228**	PS 44:2	**HL303**	ST 28:1;O4
**HL4**	CAR 16:0;O	**HL79**	LPA 19:0	**HL154**	PC 42:3	**HL229**	PS 44:4	**HL304**	ST 28:1;O5
**HL5**	CAR 16:1;O	**HL80**	LPA 20:1	**HL155**	PC 42:4	**HL230**	PS O-41:1	**HL305**	ST 28:1;O9
**HL6**	CE 22:4	**HL81**	LPA 20:2	**HL156**	PC 42:5	**HL231**	PS O-42:0	**HL306**	ST 28:2;O3
**HL7**	CE 24:1	**HL82**	LPA 20:3	**HL157**	PC 42:6	**HL232**	PS O-42:1	**HL307**	ST 28:2;O4
**HL8**	CE 26:0	**HL83**	LPA 20:4	**HL158**	PC 44:1	**HL233**	PS O-42:2	**HL308**	ST 28:2;O5
**HL9**	CL 74:2	**HL84**	LPA 22:2	**HL159**	PC 44:3	**HL234**	PS O-42:3	**HL309**	ST 28:2;O7
**HL10**	Cer 44:0;O4	**HL85**	LPA O-16:0	**HL160**	PC 44:5	**HL235**	PS O-42:7	**HL310**	ST 28:3;O2
**HL11**	Cer 44:1;O4	**HL86**	LPA O-16:1	**HL161**	PC O-13:1	**HL236**	SHexCer 42:1;O2	**HL311**	ST 28:3;O3
**HL12**	Cer 46:0;O2	**HL87**	LPA O-18:0	**HL162**	PC O-34:0	**HL237**	SM 36:0;O2	**HL312**	ST 28:3;O4
**HL13**	CerP 26:1;O2	**HL88**	LPA O-18:1	**HL163**	PC O-39:2	**HL238**	SM 36:1;O2	**HL313**	ST 28:3;O5
**HL14**	CerPE 39:1;O2	**HL89**	LPA O-20:0	**HL164**	PC O-40:4	**HL239**	SM 40:0;O2	**HL314**	ST 28:5;O3
**HL15**	CoA 10:2;O	**HL90**	LPC O-10:1	**HL165**	PC O-42:0	**HL240**	SM 40:1;O2	**HL315**	ST 28:5;O7
**HL16**	CoA 26:0;O	**HL91**	LPC O-14:1	**HL166**	PC O-42:1	**HL241**	SM 41:1;O2	**HL316**	ST 28:6;O3
**HL17**	CoA 6:0	**HL92**	LPC O-18:2	**HL167**	PC O-42:2	**HL242**	SM 41:2;O2	**HL317**	ST 28:6;O5
**HL18**	DG 26:0	**HL93**	LPE 14:1	**HL168**	PC O-42:6	**HL243**	SM 42:0;O2	**HL318**	ST 28:6;O6
**HL19**	DG 30:0	**HL94**	LPE 16:1	**HL169**	PC O-42:7	**HL244**	SM 42:1;O2	**HL319**	ST 29:2;O4
**HL20**	DG 30:3	**HL95**	LPE 17:2	**HL170**	PE 12:0	**HL245**	ST 19:0;O2;GlcA	**HL320**	ST 29:3;O3
**HL21**	DG 32:5	**HL96**	LPG 14:0	**HL171**	PE 44:0	**HL246**	ST 19:0;O3;GlcA	**HL321**	ST 29:3;O4
**HL22**	DG 33:4	**HL97**	LPG 14:1	**HL172**	PE 44:1	**HL247**	ST 19:1;O2;GlcA	**HL322**	ST 29:3;O6
**HL23**	DG 44:0	**HL98**	LPG 15:0	**HL173**	PE 44:2	**HL248**	ST 19:1;O3	**HL323**	ST 29:4;O3
**HL24**	DG 44:1	**HL99**	LPG 15:1	**HL174**	PE 44:6	**HL249**	ST 19:2;O2;GlcA	**HL324**	ST 29:4;O4
**HL25**	DG 44:2	**HL100**	LPG 16:1	**HL175**	PE 44:8	**HL250**	ST 19:2;O3	**HL325**	ST 29:5;O5
**HL26**	DG O-32:2	**HL101**	LPG 17:0	**HL176**	PE O-36:4	**HL251**	ST 19:3;O2;GlcA	**HL326**	ST 29:5;O6
**HL27**	DGDG 36:8	**HL102**	LPG 17:1	**HL177**	PE O-37:0	**HL252**	ST 19:3;O3	**HL327**	ST 29:6;O4
**HL28**	FA 16:0;O3	**HL103**	LPG 17:2	**HL178**	PE O-42:2	**HL253**	ST 20:2;O2	**HL328**	ST 29:6;O8
**HL29**	FA 16:1;O3	**HL104**	LPG 18:0	**HL179**	PG 16:0	**HL254**	ST 20:3;O2	**HL329**	ST 29:7;O5
**HL30**	FA 16:2;O3	**HL105**	LPG 18:1	**HL180**	PG 20:1;O	**HL255**	ST 21:0;O2;GlcA	**HL330**	ST 30:3;O5

**HL31**	FA 18:3;O7	**HL106**	LPG 18:3	**HL181**	PG 41:0	**HL256**	ST 21:2;O3;S	**HL331**	ST 30:3;O7
**HL32**	FA 19:4;O	**HL107**	LPG O-16:0	**HL182**	PG 41:2	**HL257**	ST 23:0;O5	**HL332**	ST 30:4;O3
**HL33**	FA 20:5	**HL108**	LPS 13:0	**HL183**	PG 41:6	**HL258**	ST 23:1;O5	**HL333**	ST 30:4;O7
**HL34**	FA 20:6	**HL109**	LPS 15:0	**HL184**	PG 42:0	**HL259**	ST 24:0;O4	**HL334**	ST 30:5;O6
**HL35**	FA 22:4;O4	**HL110**	LPS 15:1	**HL185**	PG 42:1	**HL260**	ST 24:0;O4;S	**HL335**	ST 30:5;O7
**HL36**	FA 22:5;O4	**HL111**	LPS 16:0	**HL186**	PG 42:2	**HL261**	ST 24:1;O2;Tau	**HL336**	ST 30:6;O6
**HL37**	FA 23:3;O3	**HL112**	LPS 16:1	**HL187**	PG 42:4	**HL262**	ST 24:1;O3;S	**HL337**	ST 30:6;O7
**HL38**	FA 23:3;O5	**HL113**	LPS O-15:1;O	**HL188**	PG 43:6	**HL263**	ST 24:1;O4;S	**HL338**	ST 31:7;O5
**HL39**	FA 23:3;O6	**HL114**	LPS O-16:0	**HL189**	PG 44:5	**HL264**	ST 24:1;O5	**HL339**	TG 41:2
**HL40**	FA 23:4;O4	**HL115**	LPS O-16:1	**HL190**	PG 44:6	**HL265**	ST 24:1;O5;S	**HL340**	TG 43:2
**HL41**	FA 24:0;O	**HL116**	LPS O-16:1;O	**HL191**	PG O-34:0	**HL266**	ST 24:1;O6	**HL341**	TG 45:0
**HL42**	FA 24:5;O6	**HL117**	M(IP)2C 36:0;O4	**HL192**	PG O-38:3	**HL267**	ST 24:2;O6	**HL342**	TG 45:1
**HL43**	FA 25:1	**HL118**	MG 20:0	**HL193**	PG O-39:1	**HL268**	ST 24:4;O5	**HL343**	TG 47:2
**HL44**	FA 25:2	**HL119**	MG 20:4;O	**HL194**	PG O-40:1	**HL269**	ST 24:5;O6	**HL344**	TG 47:3
**HL45**	FA 25:5;O6	**HL120**	MG 22:6	**HL195**	PG O-40:2	**HL270**	ST 25:1;O5	**HL345**	TG 48:1
**HL46**	FA 25:8;O5	**HL121**	MG O-19:0;O	**HL196**	PG O-42:2	**HL271**	ST 25:5;O8	**HL346**	TG 49:3
**HL47**	FA 26:1	**HL122**	MG O-20:0;O	**HL197**	PG O-42:4	**HL272**	ST 26:0;O	**HL347**	TG 50:4
**HL48**	FA 26:2	**HL123**	MG O-20:1;O	**HL198**	PG O-42:5	**HL273**	ST 26:0;O5	**HL348**	TG 50:5
**HL49**	FA 26:2;O4	**HL124**	MG O-21:0;O	**HL199**	PI 34:2	**HL274**	ST 26:0;O6	**HL349**	TG 50:6
**HL50**	FA 26:5;O2	**HL125**	MG O-21:1;O	**HL200**	PI 34:4	**HL275**	ST 26:1;O5	**HL350**	TG 50:8
**HL51**	FA 26:6;O4	**HL126**	MG O-23:6;O	**HL201**	PI 36:1	**HL276**	ST 26:2;O4	**HL351**	TG 50:9
**HL52**	FA 27:2	**HL127**	MIPC 34:0;O2	**HL202**	PI 37:0	**HL277**	ST 26:3;O3	**HL352**	TG 51:12;O2
**HL53**	FA 27:4	**HL128**	MIPC 34:0;O3	**HL203**	PI 37:1	**HL278**	ST 26:4;O3	**HL353**	TG 51:6
**HL54**	FA 28:6	**HL129**	MIPC 34:0;O4	**HL204**	PI 38:3	**HL279**	ST 26:5;O8	**HL354**	TG 52:7
**HL55**	FA 28:7	**HL130**	MIPC 36:0;O2	**HL205**	PI 38:7	**HL280**	ST 27:0	**HL355**	TG 52:9
**HL56**	FA 30:1;O2	**HL131**	MIPC 36:0;O3	**HL206**	PI 38:9	**HL281**	ST 27:0;O5	**HL356**	TG 53:10
**HL57**	FA 32:0;O	**HL132**	MIPC 38:0;O4	**HL207**	PI 39:6	**HL282**	ST 27:0;O8	**HL357**	TG 53:14;O2
**HL58**	FA 33:1;O	**HL133**	MIPC 40:0;O2	**HL208**	PI 39:8	**HL283**	ST 27:0;O9	**HL358**	TG 53:18;O3
**HL59**	FA 36:0	**HL134**	MIPC 40:0;O3	**HL209**	PI 42:1	**HL284**	ST 27:1;O	**HL359**	TG 53:8
**HL60**	FA 45:0	**HL135**	MIPC 42:0;O4	**HL210**	PI 42:7	**HL285**	ST 27:1;O2	**HL360**	TG 55:11
**HL61**	FAHFA 32:1;O	**HL136**	MIPC 44:0;O2	**HL211**	PI 43:4	**HL286**	ST 27:1;O3	**HL361**	TG 63:10
**HL62**	FAHFA 33:3;O	**HL137**	MIPC 46:0;O3	**HL212**	PI 43:6	**HL287**	ST 27:1;O4;S	**HL362**	TG 63:8
**HL63**	FAHFA 35:3;O	**HL138**	NAE 22:0	**HL213**	PI 44:12	**HL288**	ST 27:1;O6	**HL363**	TG 65:4

**HL64**	FAHFA 37:3;O	**HL139**	NAT 19:0	**HL214**	PI O-36:2	**HL289**	ST 27:1;O;S	**HL364**	TG 66:11
**HL65**	FAHFA 50:2;O	**HL140**	PA 20:0	**HL215**	PI O-37:2	**HL290**	ST 27:2;O	**HL365**	WE 22:1;O4
**HL66**	FAHFA 52:2;O	**HL141**	PA 20:1;O	**HL216**	PI O-37:3	**HL291**	ST 27:2;O3	**HL366**	WE 24:1;O4
**HL67**	Hex2Cer 34:0;O2	**HL142**	PA 24:3;O2	**HL217**	PI O-38:0	**HL292**	ST 27:2;O4	**HL367**	WE 24:2;O4
**HL68**	Hex2Cer 34:1;O2	**HL143**	PA 40:0	**HL218**	PI O-38:1	**HL293**	ST 27:2;O5	**HL368**	WE 25:1
**HL69**	Hex2Cer 36:0;O2	**HL144**	PA 42:1	**HL219**	PI O-40:6	**HL294**	ST 27:2;O8	**HL369**	WE 25:3
**HL70**	HexCer 39:1;O2	**HL145**	PA 43:1	**HL220**	PIP 34:1	**HL295**	ST 27:2;O9	**HL370**	WE 26:1
**HL71**	HexCer 40:0;O4	**HL146**	PA 44:1	**HL221**	PIP 37:4	**HL296**	ST 27:3;O4	**HL371**	WE 26:2
**HL72**	HexCer 43:1;O4	**HL147**	PA O-40:2	**HL222**	PS 40:0	**HL297**	ST 27:3;O5	**HL372**	WE 34:3
**HL73**	IPC 40:0;O2	**HL148**	PA O-40:4	**HL223**	PS 40:1	**HL298**	ST 27:4;O4	**HL373**	WE 36:0
**HL74**	IPC 40:1;O2	**HL149**	PC 14:0	**HL224**	PS 41:0	**HL299**	ST 27:4;O5	**HL374**	WE 38:7
**HL75**	IPC 44:0;O3	**HL150**	PC 41:0	**HL225**	PS 41:1	**HL300**	ST 27:4;O6	**HL375**	WE 44:0

HL, honey lipid. For details about lipid abbreviations, please refer to LIPID MAPS® database.

Table 4S. Identified Lipids in Bee Pollen Samples and Their Associated Codes.

Code	Lipid	Code	Lipid	Code	Lipid	Code	Lipid	Code	Lipid
**PL1**	CAR 16:0;O	**PL69**	LPA 20:2	**PL137**	PC 42:3	**PL205**	PI O-37:1	**PL273**	ST 28:1;O5
**PL2**	CAR 18:0;O	**PL70**	LPA 20:3	**PL138**	PC 42:4	**PL206**	PI O-37:2	**PL274**	ST 28:1;O9
**PL3**	CE 22:4	**PL71**	LPA 22:1	**PL139**	PC 42:5	**PL207**	PI O-38:0	**PL275**	ST 28:2;O3
**PL4**	CE 22:5	**PL72**	LPA 22:2	**PL140**	PC 44:3	**PL208**	PI O-38:1	**PL276**	ST 28:2;O4
**PL5**	CE 24:1	**PL73**	LPA 22:6	**PL141**	PC 44:5	**PL209**	PI O-40:6	**PL277**	ST 28:2;O7
**PL6**	CE 26:0	**PL74**	LPA O-18:0	**PL142**	PC O-12:1	**PL210**	PIP 34:1	**PL278**	ST 28:2;O;S
**PL7**	CL 72:2	**PL75**	LPA O-20:0	**PL143**	PC O-13:1	**PL211**	PIP 37:4	**PL279**	ST 28:3;O2
**PL8**	CL 74:4	**PL76**	LPA O-20:1	**PL144**	PC O-33:3	**PL212**	PS 40:0	**PL280**	ST 28:3;O3
**PL9**	Cer 44:0;O4	**PL77**	LPC 15:1	**PL145**	PC O-34:0	**PL213**	PS 40:1	**PL281**	ST 28:3;O4
**PL10**	Cer 44:1;O4	**PL78**	LPC O-14:0	**PL146**	PC O-39:0	**PL214**	PS 41:0	**PL282**	ST 28:3;O5
**PL11**	Cer 46:0;O2	**PL79**	LPC O-14:1	**PL147**	PC O-42:0	**PL215**	PS 41:1	**PL283**	ST 28:4;O3
**PL12**	CerP 26:1;O2	**PL80**	LPC O-18:2	**PL148**	PC O-42:1	**PL216**	PS 44:0	**PL284**	ST 28:5;O3
**PL13**	CerPE 36:0;O3	**PL81**	LPC O-19:0	**PL149**	PC O-42:2	**PL217**	PS 44:2	**PL285**	ST 28:5;O7
**PL14**	CerPE 37:2;O2	**PL82**	LPC O-20:1	**PL150**	PC O-42:4	**PL218**	PS 44:4	**PL286**	ST 28:6;O3
**PL15**	CerPE 39:2;O3	**PL83**	LPE 15:1	**PL151**	PC O-42:6	**PL219**	PS O-41:1	**PL287**	ST 28:6;O6
**PL16**	CoA 22:0	**PL84**	LPE 16:1	**PL152**	PC O-42:7	**PL220**	PS O-42:0	**PL288**	ST 28:6;O7
**PL17**	CoA 22:2	**PL85**	LPE 18:1	**PL153**	PE 12:0	**PL221**	PS O-42:1	**PL289**	ST 29:3;O3
**PL18**	CoA 26:0	**PL86**	LPE 18:2	**PL154**	PE 35:4	**PL222**	PS O-42:2	**PL290**	ST 29:3;O4
**PL19**	CoA 26:1	**PL87**	LPE 18:3	**PL155**	PE 44:0	**PL223**	PS O-42:7	**PL291**	ST 29:3;O5
**PL20**	CoA 2:0	**PL88**	LPG 15:0	**PL156**	PE 44:1	**PL224**	SM 34:2;O2	**PL292**	ST 29:3;O6
**PL21**	DG 30:0	**PL89**	LPG 15:1	**PL157**	PE 44:2	**PL225**	SM 40:0;O2	**PL293**	ST 29:3;O7
**PL22**	DG 30:3	**PL90**	LPG 16:1	**PL158**	PE 44:6	**PL226**	SM 41:1;O2	**PL294**	ST 29:4;O4
**PL23**	DG 31:2	**PL91**	LPG 17:0	**PL159**	PE 44:7	**PL227**	SM 42:0;O2	**PL295**	ST 29:5;O5
**PL24**	DG 32:5	**PL92**	LPG 17:1	**PL160**	PE 44:8	**PL228**	ST 18:4;O3;S	**PL296**	ST 29:5;O6
**PL25**	DG 33:4	**PL93**	LPG 17:2	**PL161**	PE O-36:3	**PL229**	ST 19:0;O2;GlcA	**PL297**	ST 29:7;O5
**PL26**	DG 42:4	**PL94**	LPG 18:0	**PL162**	PE O-37:0	**PL230**	ST 19:0;O3;GlcA	**PL298**	ST 30:2;O4
**PL27**	DG 44:0	**PL95**	LPG 18:1	**PL163**	PE O-42:0	**PL231**	ST 19:1;O2;GlcA	**PL299**	ST 30:3;O3
**PL28**	DG 44:2	**PL96**	LPG 18:3	**PL164**	PG 16:0	**PL232**	ST 19:2;O2;GlcA	**PL300**	ST 30:3;O5
**PL29**	FA 16:0;O3	**PL97**	LPG O-18:0	**PL165**	PG 20:1;O	**PL233**	ST 19:2;O3	**PL301**	ST 30:4;O3
**PL30**	FA 16:1;O3	**PL98**	LPG O-18:1	**PL166**	PG 20:1;O2	**PL234**	ST 19:3;O3	**PL302**	ST 30:4;O7

**PL31**	FA 16:2;O3	**PL99**	LPI 17:1	**PL167**	PG 33:0	**PL235**	ST 19:4;O3	**PL303**	ST 30:5;O6
**PL32**	FA 19:7;O	**PL100**	LPI 17:2	**PL168**	PG 35:1	**PL236**	ST 20:2;O2	**PL304**	ST 30:5;O7
**PL33**	FA 20:5	**PL101**	LPS 15:0	**PL169**	PG 35:2	**PL237**	ST 23:0;O5	**PL305**	ST 30:6;O6
**PL34**	FA 23:3;O3	**PL102**	LPS 15:1	**PL170**	PG 38:0	**PL238**	ST 24:0;O4;S	**PL306**	ST 30:6;O7
**PL35**	FA 23:3;O5	**PL103**	LPS 16:1	**PL171**	PG 41:0	**PL239**	ST 24:1;O6	**PL307**	ST 31:7;O5
**PL36**	FA 23:3;O6	**PL104**	LPS 17:1	**PL172**	PG 41:2	**PL240**	ST 24:5;O6	**PL308**	TG 41:1
**PL37**	FA 23:4;O4	**PL105**	LPS O-15:1;O	**PL173**	PG 42:0	**PL241**	ST 25:1;O5	**PL309**	TG 41:2
**PL38**	FA 24:0;O	**PL106**	LPS O-16:0	**PL174**	PG 42:1	**PL242**	ST 26:0;O	**PL310**	TG 42:4
**PL39**	FA 24:5;O6	**PL107**	LPS O-16:1	**PL175**	PG 42:3	**PL243**	ST 26:0;O6	**PL311**	TG 43:0
**PL40**	FA 25:5;O6	**PL108**	MG O-19:0;O	**PL176**	PG 43:0	**PL244**	ST 26:2;O4	**PL312**	TG 43:1
**PL41**	FA 26:2;O4	**PL109**	MG O-21:1;O	**PL177**	PG 43:6	**PL245**	ST 26:5;O8	**PL313**	TG 43:2
**PL42**	FA 26:5;O2	**PL110**	MG O-23:6;O	**PL178**	PG 44:4	**PL246**	ST 27:0	**PL314**	TG 45:0
**PL43**	FA 26:6;O4	**PL111**	MIPC 34:0;O2	**PL179**	PG 44:5	**PL247**	ST 27:0;O	**PL315**	TG 45:1
**PL44**	FA 28:2;O	**PL112**	MIPC 34:0;O4	**PL180**	PG 44:6	**PL248**	ST 27:0;O5	**PL316**	TG 47:2
**PL45**	FA 28:6	**PL113**	NAT 22:1	**PL181**	PG O-33:0	**PL249**	ST 27:0;O6	**PL317**	TG 48:1
**PL46**	FA 30:1;O2	**PL114**	NAT 23:1	**PL182**	PG O-33:1	**PL250**	ST 27:0;O8	**PL318**	TG 48:3
**PL47**	FA 32:0;O	**PL115**	PA 20:1;O	**PL183**	PG O-34:0	**PL251**	ST 27:0;O9	**PL319**	TG 49:6
**PL48**	FA 32:6;O2	**PL116**	PA 20:1;O2	**PL184**	PG O-35:3	**PL252**	ST 27:1	**PL320**	TG 50:5
**PL49**	FA 33:1;O	**PL117**	PA 23:2;O3	**PL185**	PG O-39:1	**PL253**	ST 27:1;O	**PL321**	TG 50:6
**PL50**	FA 42:0;O	**PL118**	PA 24:3;O2	**PL186**	PG O-40:1	**PL254**	ST 27:1;O4;S	**PL322**	TG 50:9
**PL51**	FA 43:0	**PL119**	PA 26:3;O3	**PL187**	PG O-42:0	**PL255**	ST 27:1;O5	**PL323**	TG 51:12;O2
**PL52**	FAHFA 32:1;O	**PL120**	PA 39:4	**PL188**	PG O-42:2	**PL256**	ST 27:1;O6	**PL324**	TG 52:10
**PL53**	FAHFA 33:3;O	**PL121**	PA 40:0	**PL189**	PG O-42:5	**PL257**	ST 27:1;O;S	**PL325**	TG 52:7
**PL54**	FAHFA 37:3;O	**PL122**	PA 42:1	**PL190**	PI 37:0	**PL258**	ST 27:2;O	**PL326**	TG 53:10
**PL55**	FAHFA 48:2;O	**PL123**	PA 43:1	**PL191**	PI 39:6	**PL259**	ST 27:2;O2	**PL327**	TG 53:18;O3
**PL56**	FAHFA 52:2;O	**PL124**	PA 44:0	**PL192**	PI 39:8	**PL260**	ST 27:2;O3	**PL328**	TG 53:2
**PL57**	Hex2Cer 36:0;O2	**PL125**	PA 44:1	**PL193**	PI 40:1	**PL261**	ST 27:2;O4;S	**PL329**	TG 53:8
**PL58**	HexCer 35:3;O3	**PL126**	PA O-37:0	**PL194**	PI 40:7	**PL262**	ST 27:2;O5	**PL330**	TG 54:6
**PL59**	HexCer 38:0;O2	**PL127**	PA O-40:2	**PL195**	PI 42:0	**PL263**	ST 27:2;O9	**PL331**	TG 63:10
**PL60**	HexCer 39:1;O2	**PL128**	PC 12:0	**PL196**	PI 42:1	**PL264**	ST 27:3;O4	**PL332**	TG 63:8
**PL61**	HexCer 42:0;O2	**PL129**	PC 14:0	**PL197**	PI 42:2	**PL265**	ST 27:3;O5	**PL333**	TG 64:15
**PL62**	HexCer 43:1;O4	**PL130**	PC 32:4	**PL198**	PI 42:5	**PL266**	ST 27:4;O4	**PL334**	TG 64:17
**PL63**	IPC 40:0;O2	**PL131**	PC 41:0	**PL199**	PI 42:7	**PL267**	ST 27:4;O5	**PL335**	WE 24:2;O4

**PL64**	IPC 42:0;O4	**PL132**	PC 41:1	**PL200**	PI 43:4	**PL268**	ST 27:4;O6	**PL336**	WE 34:3
**PL65**	IPC 44:0;O3	**PL133**	PC 41:2	**PL201**	PI 43:6	**PL269**	ST 27:5;O3	**PL337**	WE 38:7
**PL66**	LPA 16:1	**PL134**	PC 41:6	**PL202**	PI 44:10	**PL270**	ST 28:0;O		
**PL67**	LPA 18:0	**PL135**	PC 41:7	**PL203**	PI 44:12	**PL271**	ST 28:0;O5		
**PL68**	LPA 20:1	**PL136**	PC 42:2	**PL204**	PI 44:7	**PL272**	ST 28:1;O4		

PL, bee pollen lipid. For details about lipid abbreviations, please refer to LIPID MAPS® database.

Table 5S. Classification of Honey Samples According to Botanical Origin Using Principal Component Analysis.

From	Chestnut	Heather	Lavender	Multifloral	Rapeseed	Sainfoin	Trifolium	Total
**Chestnut**	2<br>100.00	0<br>0.00	0<br>0.00	0<br>0.00	0<br>0.00	0<br>0.00	0<br>0.00	2<br>100.00
**Heather**	0<br>0.00	2<br>100.00	0<br>0.00	0<br>0.00	0<br>0.00	0<br>0.00	0<br>0.00	2<br>100.00
**Lavender**	0<br>0.00	0<br>0.00	2<br>100.00	0<br>0.00	0<br>0.00	0<br>0.00	0<br>0.00	2<br>100.00
**Multifloral**	0<br>0.00	0<br>0.00	0<br>0.00	6<br>100.00	0<br>0.00	0<br>0.00	0<br>0.00	6<br>100.00
**Rapeseed**	0<br>0.00	0<br>0.00	0<br>0.00	0<br>0.00	1<br>100.00	0<br>0.00	0<br>0.00	1<br>100.00
**Sainfoin**	0<br>0.00	0<br>0.00	0<br>0.00	0<br>0.00	0<br>0.00	1<br>100.00	0<br>0.00	1<br>100.00
**Trifolium**	0<br>0.00	0<br>0.00	0<br>0.00	0<br>0.00	0<br>0.00	0<br>0.00	1<br>100.00	1<br>100.00
**Total**	2<br>13.33	2<br>13.33	2<br>13.33	6<br>40.00	1<br>6.67	1<br>6.67	1<br>6.67	15<br>100.00

Table 6S. Classification of Honey Samples According to Geographical Origin Using Principal Component Analysis.

From	Castilla La Mancha	Castilla y León	Spain	Total
**Castilla La Mancha**	3<br>100.00	0<br>0.00	0<br>0.00	3<br>100.00
**Castilla y León**	0<br>0.00	11<br>100.00	0<br>0.00	11<br>100.00
**Spain**	0<br>0.00	0<br>0.00	1<br>100.00	1<br>100.00
**Total**	3<br>20.00	11<br>73.33	1<br>6.67	15<br>100.00

Table 7S. Classification of Bee Pollen Samples According to Botanical Origin Using Principal Component Analysis.

From	Chestnut	Multifloral	Total
**Chestnut**	2<br>66.67	1<br>33.33	3<br>100.00
**Multifloral**	1<br>10.00	9<br>90.00	10<br>100.00
**Total**	3<br>23.08	10<br>76.92	13<br>100.00

Table 8S. Classification of Bee Pollen Samples According to Geographical Origin Using Principal Component Analysis.

From	Andalucía	Castilla y León	Galicia	Spain	Total
**Andalucía**	1<br>100.00	0<br>0.00	0<br>0.00	0<br>0.00	1<br>100.00
**Castilla y León**	0<br>0.00	1<br>100.00	0<br>0.00	0<br>0.00	1<br>100.00
**Galicia**	0<br>0.00	0<br>0.00	3<br>100.00	0<br>0.00	3<br>100.00
**Spain**	0<br>0.00	0<br>0.00	0<br>0.00	8<br>100.00	8<br>100.00
**Total**	1<br>7.69	1<br>7.69	3<br>23.08	8<br>61.54	13<br>100.00

Table 9S. Classification of Bee Pollen Samples According to Geographical Origin Within the Same Botanical Origin Using Principal Component Analysis.

From	Andalucía	Castilla y León	Galicia	Spain	Total
**Andalucía**	1<br>100.00	0<br>0.00	0<br>0.00	0<br>0.00	1<br>100.00
**Castilla y León**	0<br>0.00	1<br>100.00	0<br>0.00	0<br>0.00	1<br>100.00
**Galicia**	0<br>0.00	0<br>0.00	2<br>100.00	0<br>0.00	2<br>100.00
**Spain**	0<br>0.00	0<br>0.00	0<br>0.00	6<br>100.00	6<br>100.00
**Total**	1<br>7.69	1<br>7.69	2<br>23.08	6<br>61.54	10<br>100.00

Figure 1S. Comparative MALDI-TOF Mass Spectra of the Best MALDI Matrices Used in This Study.

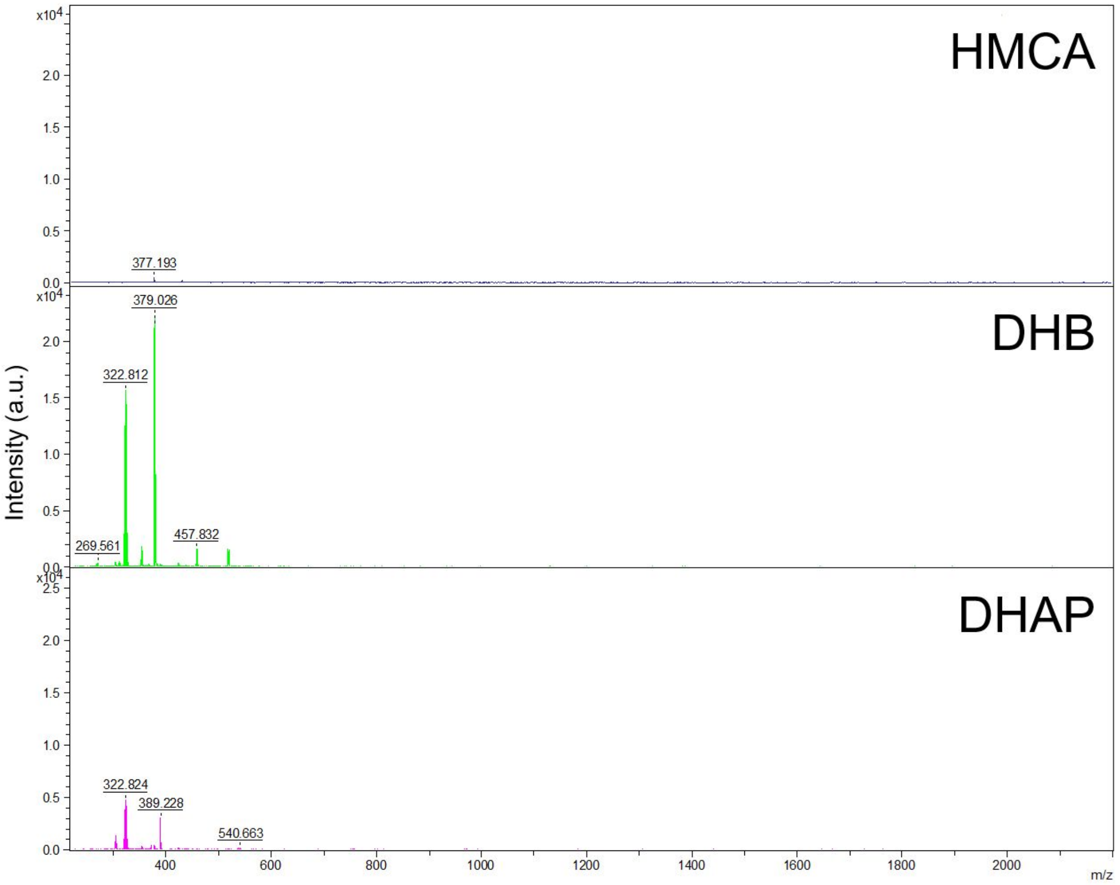

Figure 2S. MALDI-TOF Mass Spectra Showing Main Lipids Detected in Honey With 50% Laser Intensity Using a Sample:Matrix:Agent Ratio of 1:1:0.25. MALDI Matrix is DHAP and Ionizing Agent is AgTFA in THF (10 mg mL^{-1}).

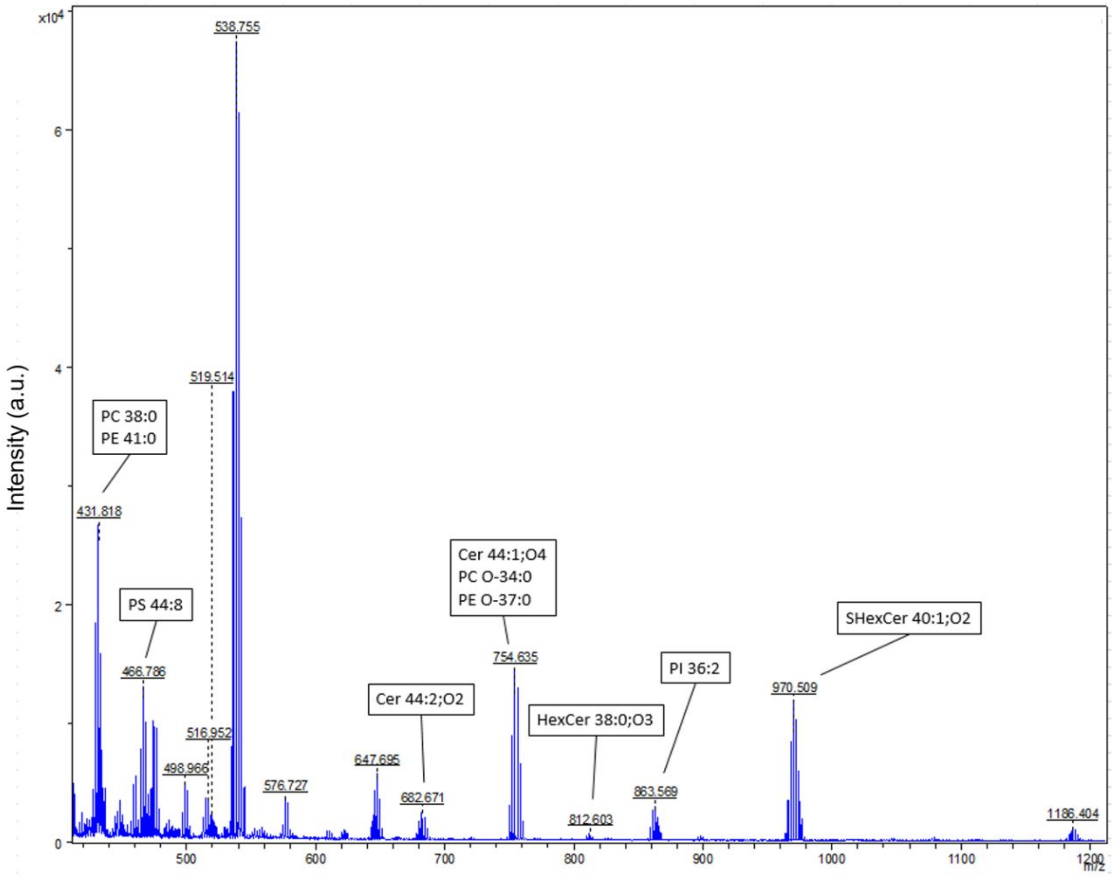

Figure 3S. Comparative MALDI-TOF Mass Spectra of Bee Pollen Samples Acquired at Different Laser Intensities.

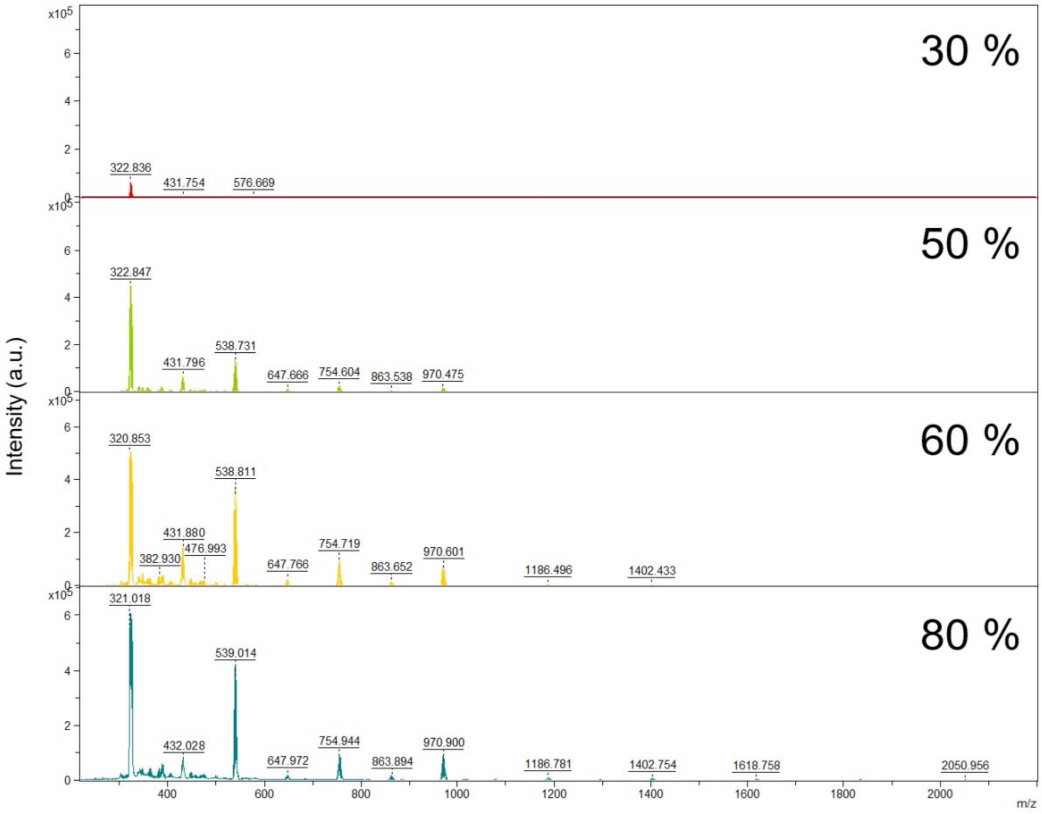

Figure 4S. Metrics Used for Green and Blue Assessment of the Proposed Methods: A) AGREE for Honey, B) AGREE for Bee Pollen, C) MoGAPI Score for Honey and Bee Pollen, D) BAGI Score for Honey and Bee Pollen.

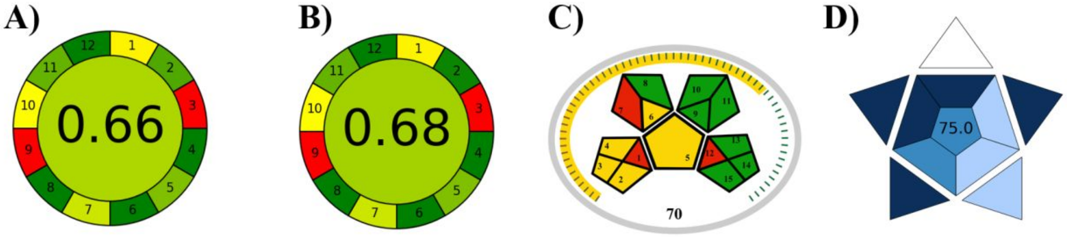

Supplement: Supplementary file 1 [file as5c00883_si_001.pdf]
